# Multiomic Analyses Reveal the Molecular Mechanisms of Arid Adaptation in a Desert Rodent Species

**DOI:** 10.1093/molbev/msaf230

**Published:** 2025-09-17

**Authors:** Shuai Yuan, Rong Zhang, Yongling Jin, Xin Li, Linlin Li, Dong Zhang, Yu Ling, Kaijian Zhang, Xiaodong Wu, Xueying Zhang, Heping Fu

**Affiliations:** College of Grassland Science, Inner Mongolia Agricultural University, Inner Mongolia, China; State Key Laboratory of Animal Biodiversity Conservation and Integrated Pest Management, Institute of Zoology, Chinese Academy of Sciences, Beijing, China; College of Grassland Science, Inner Mongolia Agricultural University, Inner Mongolia, China; College of Grassland Science, Inner Mongolia Agricultural University, Inner Mongolia, China; College of Grassland Science, Inner Mongolia Agricultural University, Inner Mongolia, China; College of Grassland Science, Inner Mongolia Agricultural University, Inner Mongolia, China; College of Grassland Science, Inner Mongolia Agricultural University, Inner Mongolia, China; College of Grassland Science, Inner Mongolia Agricultural University, Inner Mongolia, China; Novogene Bioinformatics Institute, Beijing, China; College of Grassland Science, Inner Mongolia Agricultural University, Inner Mongolia, China; State Key Laboratory of Animal Biodiversity Conservation and Integrated Pest Management, Institute of Zoology, Chinese Academy of Sciences, Beijing, China; College of Grassland Science, Inner Mongolia Agricultural University, Inner Mongolia, China

**Keywords:** desert rodents, genome, convergent evolution, arid adaptation, metabolic water production

## Abstract

Organisms living in desert habitats face multiple simultaneous pressures, such as high temperatures and arid, and the population dynamics and community diversity of small rodents are strongly affected by climate extremes. However, the potential mechanisms by which desert rodents adapt to arid remain largely unexplored. Here, we assembled a 3.18 Gb genome, including 25,812 protein-encoding genes, for *Orientallactaga sibirica*, which is widely distributed across both arid and semihumid environments in Eurasia. *Orientallactaga sibirica* has longer ears and hind limbs to enhance heat dissipation, which may be related to the positively selected genes, such as *Fgf10*, *Fgf11*, *Hoxc4*, *Hoxd1*, and *Bmp4*. The renal transcriptome revealed increased fat and carbohydrate metabolism for metabolic water production in *O. sibirica* residing in arid habitats. Pathways such as material metabolism, oxidative stress response, osmoregulation, and water and salt reabsorption were enriched in candidate genes, such as *Avp*, *Ang*, and *Ace*, under positive selection in *O. sibirica*. Moreover, amino acid replacement was observed in the protein sequences of seven candidate genes, including *Aldh7a1*, *Lnpep*, *Wnk4*, *C1qc*, and *Awat2*, and these specific amino acid replacements of genes such as *Umod* and *Scnn1a* were related to unique osmoregulation, osmotic protection, and water retention compensation mechanisms. Water deprivation under laboratory conditions induced the upregulation of *Umod* and *Aldh7a1* expression, further supporting the results observed in the wild population. These findings demonstrate that the positively selected genes related to limb development and specific amino acid replacements in the genes *Umod* and *Scnn1a* for unique osmoregulation in the renal vascular system may contribute to arid adaptation in the desert rodent species *O. sibirica*. This study provides novel insights into the adaptive evolution of desert small mammals and can serve as a reference for future research on renal damage-related diseases, such as human kidney stones and salt-sensitive hypertension.

## Introduction

Species living in extreme environments have evolved a variety of adaptations over evolutionary time (Qiu et al. 2012; Sequencing and Consortium 2012; Zhou et al. 2016). Desert mammals have evolved multiple strategies to adapt to high temperatures and low water availability with respect to their morphological, behavioral, and physiological traits (Hill and Veghte 1976; Willmer et al. 2009; Cooper 2011; Mitchell et al. 2018). Some small rodents do not rely on evaporative cooling (Willmer et al. 2009), and their activity rhythms are altered to reduce water loss (Walsberg 2000; Cooper 2011; Monarca et al. 2019). These species also tend to produce concentrated urine (Rocha et al. 2021a) and metabolic water to reduce water loss (Willmer et al. 2009; Hyndman and Pannabecker 2015), allowing them to use water much more efficiently than their nondesert counterparts. Some species may have long limbs and altered body shapes to shed heat via conductive heat dissipation (Tseng et al. 2018; Weeks et al. 2020; Ryding et al. 2021). In the context of global warming, understanding the mechanisms by which desert rodents adapt to extreme arid environments is necessary.

Both parallel evolution, in which related species acquire similar characteristics, and convergent evolution, in which distantly related or unrelated species acquire similar characteristics, have been observed in desert mammal species (Cheviron et al. 2012; Hu and Hoekstra 2017; Ivy and Scott 2017; Storz et al. 2019). For example, *Fennecus zerda* living in desert environments has lower body weights than other foxes, which may reduce their overall energy requirements (Williams et al. 2004) and is consistent with Bergmann's rule (James 1970). *Lepus californicus* (Hill and Veghte 1976; Mitchell et al. 2018) and *Jaculus jaculus* (Cooper 2011) have long limbs for adaptation to arid environments, conforming to Allen's rule (Allen 1877). Convergent and species-specific adaptations in fat metabolism, insulin response, and arachidonic acid (AA) metabolism pathways were observed in *Camelus bactrianus* and *Cervus elaphus yarkanensis*, which may be key factors for the survival of mammals in different deserts worldwide (Sequencing and Consortium 2012; Wu et al. 2014; Yang et al. 2016; Ababaikeri et al. 2020; Tigano et al. 2020; Rocha et al. 2021b). However, the genomic traits and functional adaptations of desert rodent species remain ambiguous.

The Siberian jerboa (*Orientallactaga sibirica*), belonging to the family Dipodidae, is widely distributed in arid, semiarid, and semihumid regions in China, Kazakhstan, Kyrgyzstan, Mongolia, and other countries in Eurasia (Jiang 2015). The Siberian jerboa has long and wide ear flaps, a thin and long tail, and extremely long hind limbs. It also has a nocturnal habit of being active at night and hiding during the day, which allows it to avoid the extreme temperatures of the day and dissipate heat through increased conduction. The jerboa originally lived in humid forest environments but gradually expanded toward arid open environments as a result of geological changes (Kusano et al. 1997). This significant change in habitat may explain the large difference in the external morphology of jerboa from that of other small rodents in desert habitats.

Here, we constructed a genome-wide sequence map of *O. sibirica* via third-generation Hi-C sequencing combined with measurements of several morphological, physiological, and biochemical indices to investigate its adaptations to arid environments. We also conducted comparative genomic and convergent evolution gene expression analysis, as well as RNA sequencing on populations of *O. sibirica* across habitats with varying aridity and laboratory water-deprivation experiments, to explore the unique arid adaptation mechanisms of *O. sibirica*. We hypothesize that small rodents living in arid environments exhibit great morphological and physiological “resilience,” and certain convergences not only at the genomic level, but also at the transcriptional level. We show that positive selection genes such as fibroblast growth factor 10 (*Fgf10*), fibroblast growth factor 11 (*Fgf11*), homeobox C4 (*Hoxc4*), homeobox D1 (*Hoxxd1*), and bone morphogenetic protein 4 (*Bmp4*) may be related to enlarged limb development for heat dissipation, and the unique amino acid replacements of the genes *Umod* and *Scnn1a* may contribute to the specific osmoregulation in the renal vascular system for arid adaptation. Overall, our results enrich the genomic survey data of small wild desert rodents and genetic resources on *O. sibirica* and provide a basis for a deep understanding of arid adaptation in small mammals.

## Results

### Apparent Characteristics of Siberian Jerboa From Different Habitats

**Fig. 1.**
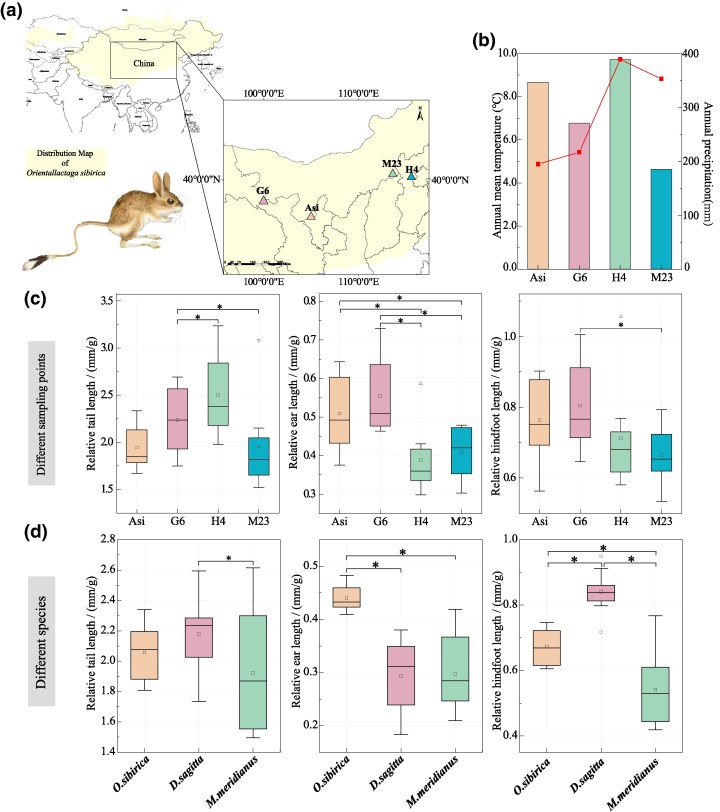
Apparent characteristics of *O. sibirica* from different sampling locations. a) Sampling locations ranging from semiarid sites in the west (Asi, Alashan, Alxa League, Inner Mongolia Autonomous Region), arid sites (G6, Minle County, Zhangye city, Gansu Province), and semihumid sites in the east (H4, Zhuolu County, Zhangjiakou city, Hebei Province and M23, Fengzhen city, Wulanchabu city, Inner Mongolia Autonomous Region). b) Annual average temperature (bar charts) and annual precipitation (the line chart) at the different sites. c) Relative tail, ear, and hindfoot length of *O. sibirica*. *n* = 10 per group. d) Relative tail, ear, and hindfoot length of three rodent species captured in the same arid habitat. *O. sibirica*, *Orientallactaga sibirica*; *D. sagitta*, *Dipus sagitta*; and *M. meridianus*, *Meriones meridianus*. *P < 0.05. *n* = 10 per group.

To determine morphological adaptations, we measured the length and weight of specific body areas of *O. sibirica* from four regions with different annual temperatures and precipitation levels (Fig. 1a and b) and compared these characteristics with those of *Meriones meridianus* and *Dipus sagitta*, which are distributed in the same region as *O. sibirica*. The relative ear length and hindfoot length were significantly greater in *O. sibirica* residing in arid areas (Asi, G6) than in humid areas (H4, M23; Fig. 1c). The relative ear length of *O. sibirica* was greater than that of the other two species (Fig. 1d). These apparent characteristics imply that *O. sibirica* evolved large ears to enhance heat dissipation and prevent heat stress in the desert.

### Genome Prediction and Genomic Comparisons

**Fig. 2.**
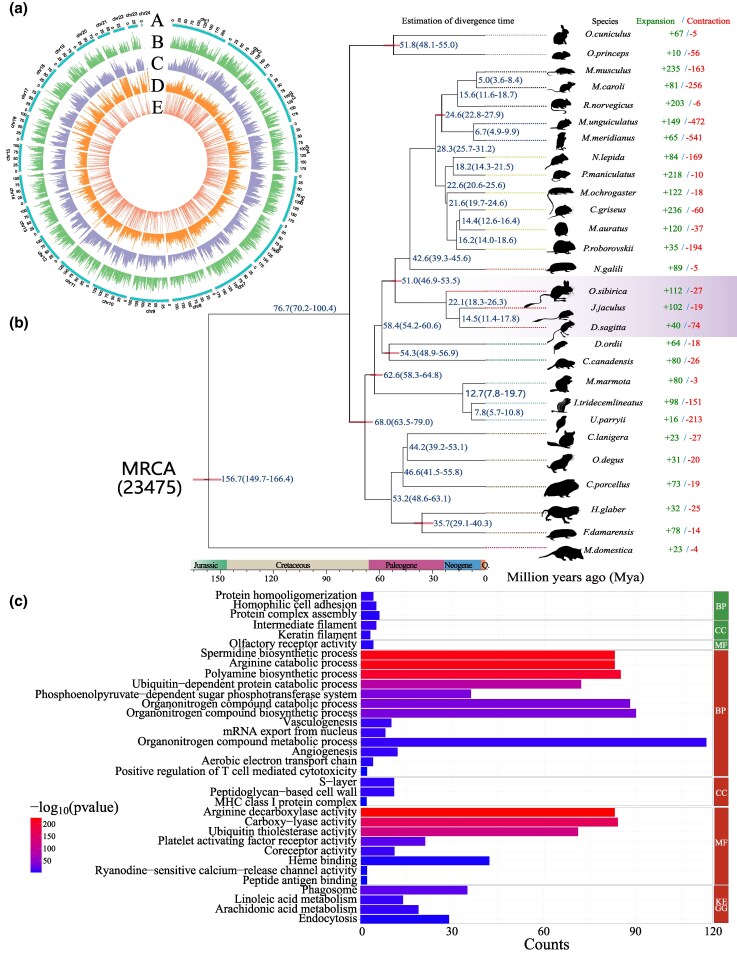
Complete genome and evolution analysis of *O. sibirica*. a) Overview of the complete genome of *O. sibirica*. A. Genomic distribution across chromosomes (1-Mb window); b) distribution of gene density; c) distribution of GC content in the chromosome genome; d) density of repeating sequences distributed throughout the genome; e) Expression quantity of each gene in the genome. b) Phylogenetic relationships between *O. sibirica* and 27 other species, on the right shows the acquired and lost gene families; the divergence time is shown on the left. c) Enrichment analysis of gained (upper half) and lost (lower half) gene families.

To investigate the genetic mechanisms underlying the adaptations of *O. sibirica* to arid environments, we generated four high-quality de novo (Hi-C) genomes. The Illumina sequence was 1,197.18 Gb, the PacBio long sequence was 289.83 Gb, the 10X genome sequence was 275.24 Gb, and the Hi-C sequence was 345.81 Gb (Tables S1 to S5 and Fig. S1). The final genome size was 3.18 Gb, with a continuous N50 of 3.77 Mb and an average coverage of 367.23% (Tables S5 and S6). The contigs of *O. sibirica* were further assembled into pseudoschizomers with a length of the entire chromosome sequence, with a scaffold N50 size of 153.97 Mb (Fig. 2a, Table S6) and a genome attachment rate of 95.26% (Table S7). Analysis of the draft genome of *O. sibirica* revealed that 93.0% of the mammalian universal benchmarking single-copy orthologs (BUSCOs) were complete, 3.5% were partially complete, and the GC content was 41.94% (Tables S8 to S11). A total of 25,812 protein-coding genes were annotated, and approximately 99.42% of the genes were functionally annotated (Table S12). Transposable elements (TEs), which are composed mainly of unknown TEs such as long terminal repeats and long interspersed nuclear elements, accounted for 50.93% of the genome assembly (Tables S9 and S10 and Figs. S2 to S5).

A phylogenetic tree was constructed to identify the evolutionary relationships of *O. sibirica* and 26 other species from Rodentia and one species from Didelphimorphia using the nucleic acid sequence of shared single-copy orthologous genes (Table S13 and Fig. S6). There were 23,479 gene families among the species, of which 2,321 single-copy families were shared by all the species (Fig. 2b). On the basis of the phylogenetic tree, the divergence time between *O. sibirica* and *J. jaculus* was ∼22.1 million years, which is consistent with paleontological evidence for *J. jaculus*. We found that *O. sibirica* acquired 112 gene families and lost 27 (Fig. 2b and c; Fig. S7 and Table S14). The expansion dataset includes gene families related to arid adaptation, such as protein metabolic process (GO:0019538), organic substance metabolic process (GO:0071704), membrane protein ectodomain proteolysis (GO:0006509), and metal ion homeostasis (GO:0055065). This set includes genes associated with water metabolism, such as acyl-CoA thioesterase 13 (*Acot13*), 3-hydroxybutyrate dehydrogenase (*Bdh2*), and peroxisome proliferator-activated receptor gamma coactivator 1 beta (*Ppargc1b*). The contraction dataset includes gene families related to arid adaptation, such as active transmembrane transporter activity (GO:0022804), regulation of systemic arterial blood pressure by renin–angiotensin (GO:0003081). This set includes genes associated with transmembrane signal receptors, such as a disintegrin and metalloproteinase domain-containing metallopeptidase 21 (*Adam21*), adenylate cyclase-activating polypeptide 1 (*Adcyap1*), and protocadherin alpha 6 (*Pcdha6*).

### Comparative Genomic Analysis Between Species From Arid and Humid Environments

**Fig. 3.**
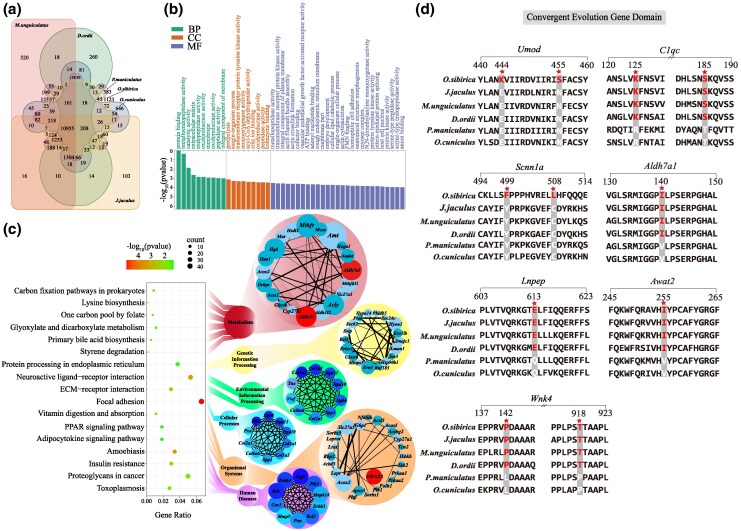
Construction and convergent evolution analysis of gene sets related to arid adaptation. a) Venn diagram of gene families of the six species (*O. sibirica*; *J. jaculus*; *M. unguiculatus*; *D. ordii*; *O. cuniculus*; *P. maniculatus*). b) GO enrichment analysis of positively selected genes. BP, biological process; CC, cellular component; MF, molecular function. c) KEGG enrichment analysis of positively selected genes and network of relationships between enriched genes. d) Differential mutations of amino acid sequences in protein-encoding genes related to arid adaptation, also showing information on sites with base replacement.

The genomes of four species residing in arid habitats (*O. sibirica*, *J. jaculus*, *Meriones unguiculatus*, and *Dipodomys ordii*) and two species residing in humid habitats (*Oryctolagus cuniculus* and *Peromyscus maniculatus*) were analyzed and compared to identify the genes involved in arid adaptations (there are 1,123 unique genes in *O. sibirica*, Fig. 3a). A total of 2,449 candidate genes were subjected to positive selection in the genome of *O. sibirica* (*P* value < 0.01, FDR < 0.05). Among those positively selected genes, 152 gene families were obtained from enrichment analysis. Pathways related to osmoregulation, such as ion transport (GO:0007250), extracellular ligand-gated ion channel activity (GO:0016491), and voltage-gated cation channel activity (GO:0030001), were significantly enriched (Fig. 3b and c and Tables S15 and S16). In addition, some pathways related to metabolic water production, such as carbohydrate metabolism (map00052) and lipid metabolism (map00564), were significantly enriched in *O. sibirica* (Tables S17 and S18). Pathways related to limb length (Table S19) and several pathways in the extended gene family related to blood pressure regulation and repair (Tables S20 and S21) were also enriched. And there is no intersection between the three datasets: expansion, contraction, and positive selection (Fig. S8).

We used the Codeml program in PAML 4.7 to reconstruct ancestral protein sequences through 7,999 orthologs among six species with parameters (RateAncestor = 1, model = 2). The convergent amino acid sites that were associated with arid adaptation were identified. We found the amino acid residues between rodents in arid habitats (*O. sibirica*, *J. jaculus*, *M. unguiculatus*, and *D. ordii*) and rodents in humid habitats (*P. maniculatus* and *O. cuniculus*) were different, in the protein sequences of genes such as leucyl and cystinyl aminopeptidase (*Lnpep* Q613E), WNK lysine deficient protein kinase 4 (*Wnk4* L142P, P918T), complement c1q c chain (*C1qc* R124K, T184S), aldehyde dehydrogenase 7 family member A1 (*Aldh7a1* V140I), and acyl-coa wax alcohol acyltransferase 2 (*Awat2* V255I) genes (Fig. 3d). Comparing the changes in ancestral amino acids across five species may provide insights into mechanisms underlying arid tolerance. Among these six species, *M. unguiculatus* is closely related to *P. maniculatus*; however, the amino acids in *P. maniculatus*, which inhabits humid environments, differ from those observed in rodents adapted to arid habitats. In addition, *O. sibirica* had unique amino acid replacements in the uromodulin (*Umod* E444K, N455S) and sodium channel epithelial 1 subunit alpha (*Scnn1a* Y499F, C508L) protein sequences, which were distinct from other species in arid habitats (Fig. 3d).

### RNA Sequencing Analysis of the Kidneys of Jerboas From Different Habitats

**Fig. 4.**
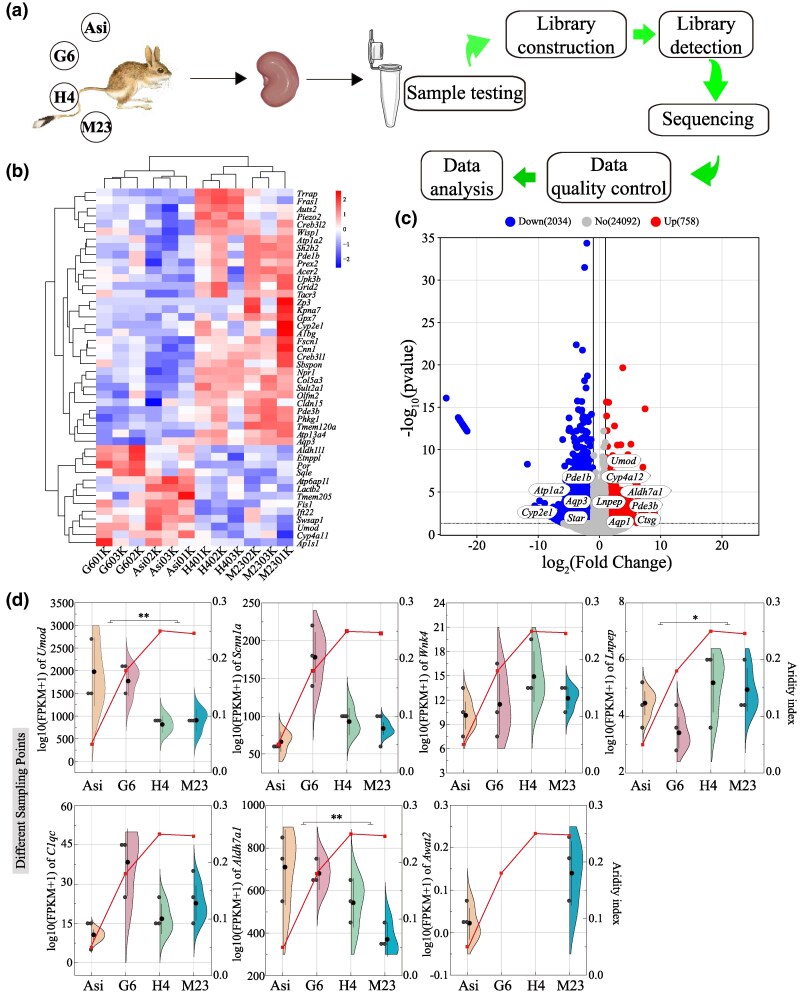
RNA sequencing analysis of the kidneys of *O. sibirica* in different habitats. a) Experimental process. b) Cluster analysis of DEGs in different habitats, where the *x* axis indicates the sample name and the *y* axis indicates the normalized fragments per kilobase of transcript per million mapped reads (FPKM) value of the DEGs. A darker red color indicates stronger expression, whereas a darker blue color indicates lower expression. c) Volcano plot comparing the DEGs in the kidneys of *O. sibirica* in arid and humid areas; left side indicates downregulated genes, and right side indicates upregulated genes. The annotated genes are key genes related to the target phenotype in the positive selection dataset. d) Expression of candidate convergent evolution genes in *O. sibirica* across habitats with varying aridity. The polyline represents AI (corresponding to the right *y* axis). Asi, Alxa League; G6, Minle County; H4, Zhuolu County; M23, Fengzhen city.

To further screen the key genes responsible for the arid adaptations of *O. sibirica*, we performed RNA sequencing of kidney tissues from the jerboas residing in four locations with varying aridity (Fig. 4a; Tables S22 and S23 and Figs. S9 and S10). The gene expression of cytochrome p450 family 4 subfamily a member 12 (*Cyp4a12*), *Umod*, *Aldh7a1*, *Lnpep*, and cathepsin g (*Ctsg*) was upregulated, whereas the expression of aquaporin 3 (*Aqp3*), and cytochrome p450 family 2 subfamily e member 1 (*Cyp2e1*) was downregulated in the rodents from arid habitats compared to those from humid habitats (Fig. 4b and c). Among the seven convergent evolution candidate genes, significant differences in the expression of *Aldh7a1*, *Umod*, and *Lnpep* were observed between the arid and humid groups. Moreover, as the aridity index (AI) decreased across the sampling sites, the expression of *Aldh7a1* and *Umod* increased, whereas the expression of *Awat2*, *Lnpep*, *C1qc*, *Scnn1a*, and *Wnk4* decreased (Fig. 4d).

**Fig. 5.**
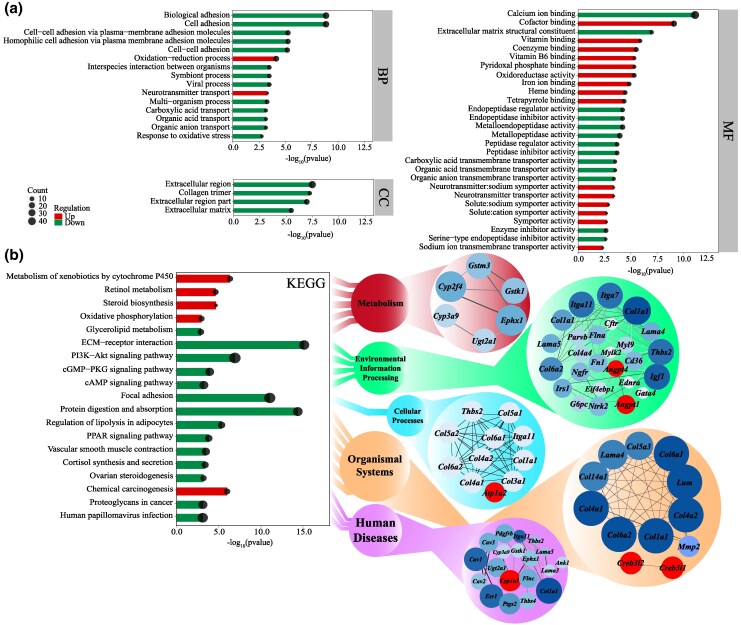
Enrichment analysis of DEGs in the kidneys of *O. sibirica* from the arid and humid habitats. a) GO enrichment analysis, BP, biological process; CC, cellular component; MF, molecular function. b) KEGG enrichment and relationship network between the enriched DEGs.

Pathways related to vascular smooth muscle contraction (rno04270), renin secretion (rno04924), AA metabolism (rno00590), cytochrome P450 (rno00982), and other pathways related to vascular contraction were significantly enriched in the differentially expressed genes (DEGs) of rodents in arid environments (Fig. 5a and b; Tables S24 and S25). Moreover, membrane protein-related pathways such as organic anion transmembrane transporter activity (GO:0008514) and sodium ion transmembrane transporter activity (GO:0015081) were significantly enriched. Additionally, the metabolic pathways related to protein digestion and absorption (rno04974) and fat digestion and absorption (rno04975) were significantly enriched (Tables S24 and S25).

### Renal Responses to Water-Deprivation Stress in *O. sibirica*

**Fig. 6.**
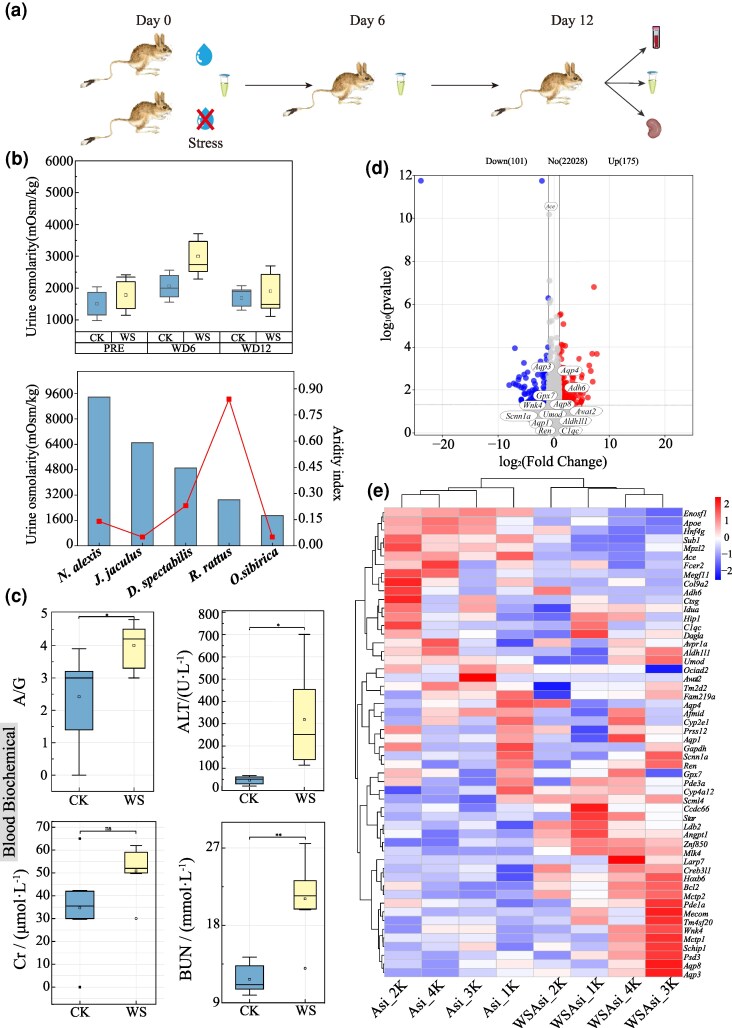
Physiological indicators and RNA sequencing analysis of the kidneys of *O. sibirica* under WS. a) Experimental design. b) Urine osmotic pressure of *O. sibirica* from the Alashan site (Asi) and comparative analysis among five rodent species (*N. alexis*, *Notomys alexis*; *J. jaculus*; *D. spectabilis*, *Dipodomys spectabilis*; *R. rattus*, *Rattus rattus*; *O. sibirica*); the red line indicates the AI. c) Effects of WS on blood biochemical indicators of *O. sibirica*, A/G, albumin/globulin ratio; BUN, urea nitrogen; ALT, alanine aminotransferase; Cr, creatinine concentration. *P < 0.05; **P < 0.01. d) Volcano plot of DEGs in the kidneys of *O. sibirica* under WS; left side indicates downregulated genes, while right side indicates upregulated genes. The annotated genes are key genes related to the target phenotype in the positive selection dataset. e) Cluster analysis of DEGs in the kidneys of *O. sibirica* between the two treatment groups; the *x* axis indicates the sample name, and the *y* axis indicates the normalized FPKM of the DEGs. Darker red indicates higher expression levels, whereas darker blue indicates lower expression levels.

To further verify the physiological and molecular responses of *O. sibirica* to water-deprivation stress (WS), we captured wild jerboas and reared them under control conditions (CK) or WS conditions (Fig. 6a). The urine concentration was measured in these two groups and compared with that of rodents collected from habitats with varying aridity (*Notomys alexis*, *J. jaculus*, *D. spectabilis*, and *Rattus rattus*) (Bozinovic and Gallardo 2006; Giorello et al. 2014). No significant difference in the maximum urine osmotic pressure was observed between the two groups of *O. sibirica* (Fig. 6b). However, the urine osmotic pressure of *O. sibirica* was lower than that from other similarly arid habitats except for *R. rattus* (Fig. 6b). The levels of blood biochemical indicators, including the albumin/globulin ratio (A/G) and urea nitrogen (BUN), alanine aminotransferase (ALT), and creatinine (Cr) concentrations, were significantly higher in the WS group than in the control group (Fig. 6c, Table S26). A total of 276 DEGs were obtained in the WS versus CON group (Table S26 and Figs. S11 to S14), with 175 upregulated genes, such as *Aqp4* and alcohol dehydrogenase 6 (Class V *Adh6*), and 101 downregulated genes, such as *Aqp3* and glutathione peroxidase 7 (*Gpx7*) (Fig. 6d and e).

**Fig. 7.**
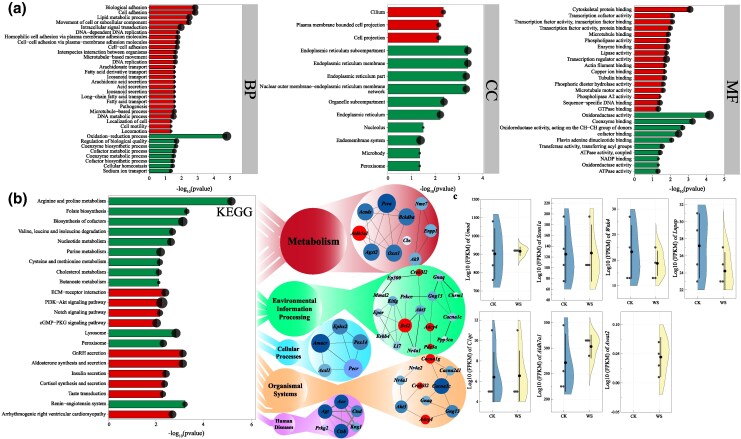
Differential gene enrichment analysis of the kidneys of *O. sibirica* under WS. a) GO enrichment analysis (BP, biological process; CC, cellular component; MF, molecular function) of DEGs and b) KEGG pathway enrichment analysis of the DEGs. c) Expression of candidate convergent evolution genes in rodents under WS. CK, control group; WS, water-deprivation stress.

The DEGs were significantly enriched in pathways such as oxidation–reduction process (GO:0055114), transmembrane transport (GO:0006855), and ion channel activity (GO:0005216) (Tables S28 and S29; Fig. 7a). Moreover, pathways such as the renin–angiotensin system (rno04614) and cytochrome P450 (rno00982) were significantly enriched. Lipid metabolism process (GO:0006629), insulin secretion (rno04911), and protein digestion and absorption (rno04974) were also significantly enriched (Tables S28 and S29). Notably, the monomer process (GO:0044699), sperm-zona pellucida binding (GO:0007339), sperm-egg recognition (GO:0035036), and single fertilization (GO:0007338) pathways were significantly enriched (Fig. 7a and b and Table S30).

Among the candidate genes related to the convergent evolution of *O. sibirica*, only *Awat2* was upregulated in the WS versus control group, whereas there were no significant differences in the expression of *Aldh7a1*, *Umod*, *Lnpep*, *C1qc*, *Scnn1a*, or *Wnk4* between the two groups (Fig. 7c).

In the cross-dataset analysis of DEGs in the kidney transcriptome of *O. sibirica* comparing wild arid_versus_humid habitats with indoor WS_versus_CK, total 47 DEGs were shared in both, such as calcium voltage-gated channel subunit alpha1h (*Cacna1h*) and transient receptor potential cation channel subfamily c member 3 (*Trpc3*), and genes such as core 1 β1, 3-galactosyltransferase 1 (*C1galt1*), wee2 oocyte meiosis inhibiting kinase (*Wee2*), zona pellucida glycoprotein 2 (*Zp2*), and zona pellucida binding protein 2 (*Zpbp2*) that are associated with sperm binding, oocyte meiosis, and acrosome reaction (Fig. S15). These shared genes were mainly enriched in fatty acid metabolic process (GO:0006631), prostanoid metabolic process (GO:0006692), AA metabolic process (GO:0019369, mmu00590), regulation of lipolysis in adipocytes (mmu04923), and VEGF signaling pathway (mmu04370) (Fig. S15b and c). Both the wide and laboratory data support that the adaptive mechanisms of *O. sibirica* in arid environments involve osmoregulation and metabolic processes.

To further verify the sequencing and analysis results, some key DEGs identified in the control versus WS group were detected by Real-Time Quantitative PCR (RT-qPCR). Compared to the control group, the WS group exhibited significant upregulation of redox-related genes lipase e, hormone sensitive type (*Lipe*), and calcium homeostasis modulator 3 (*Calhm3*), significant upregulation of genes related to the renin–angiotensin system, including *C1qc*, *Scnn1a*, and *Wnk4*; and significant upregulation of genes related to protein digestion and absorption, including collagen type VI alpha 3 chain (*Col6a3*) and chloride channel accessory 4 (*Clca4*) (Table S31 and Fig. S16). These results further confirm that *O. sibirica* upregulated the transcription processes that were involved in osmoregulation and metabolic processes under water shortage and support the sequencing and bioinformatics analysis results.

## Discussion

**Fig. 8.**
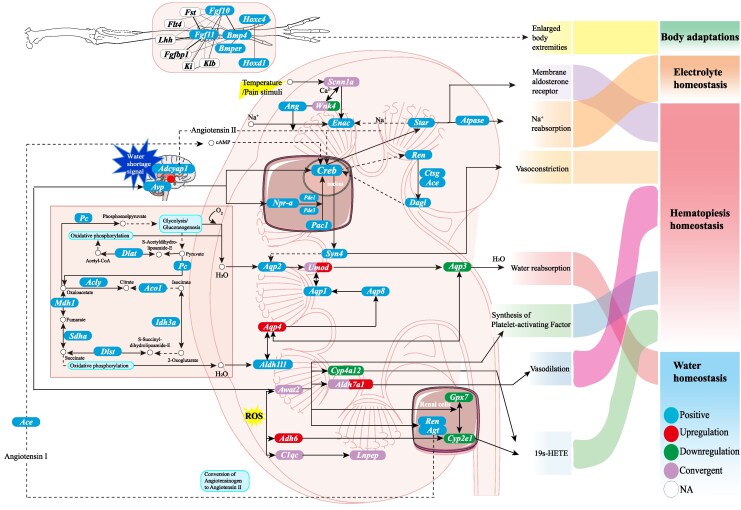
Adaptation mechanisms of *O. sibirica* in arid environments. The schematic summarizes the interactions between genes related to arid adaptation. Blue indicates positively selected genes, red indicates upregulated genes, green indicates downregulated genes, purple indicates convergent evolution genes, solid lines indicate direct interactions, and dotted lines indicate indirect interactions.

The main challenges faced by small mammals living in arid environments are to maintain a safe body temperature and to conserve water. Small mammals living in deserts often exhibit important adaptive features that have evolved independently across different species worldwide in response to similar selection pressures of extreme temperatures, aridity, and food scarcity. Allen's rule predicts that warm-blooded animals living in warmer climates will have longer appendages (limbs, tails, and ears) relative to their body size (Allen 1877). The long hind limbs and jumping movements of the jerboa (*Dipodidae* spp.) residing in deserts and arid regions are believed to have evolved to adapt to the desert environment. Moreover, organisms from colder climates tend to have larger body sizes and shorter appendages, whereas those from hotter climates have smaller body sizes and longer appendages (Cheng et al. 2018). In contrast to other sympatric desert species in arid habitats, such as *D. sagitta* and *M. meridianus*, *O. sibirica* has larger ear auricles and longer hind limbs. This morphological adaptation was supported by genomic evidence that genes related to limb development, such as *Ffg10*, *Ffg11*, *Hoxc4*, *Hoxd1*, and *Bmp4* (Sears 2008; Sears et al. 2018) were positively selected (Fig. 8). The results of the present study provide genomic evidence for the morphological adaptation to enhance heat dissipation in *O. sibirica*.

Compared to large mammals, small mammals are highly sensitive to environmental changes and evolve faster (Evans et al. 2012), and experience relatively greater environmental pressure from extreme arid desert habitats (Haugen and Vøllestad 2010). Therefore, it is not enough for small desert rodents to rely solely on morphological characteristics and behavior to cope with high temperatures and arid environment. The present data showed that the *O. sibirica* experienced rapid gene expansions such as acyl-coa thioesterase 13 (*Acot13*), anoctamin 9 (*Ano9*), 3-hydroxybutyrate dehydrogenase (*Bdh2*), peroxisome proliferator-activated receptor gamma, coactivator 1 beta (*Ppargc1b*), and cub and sushi multiple domains 3 (*Csmd3*), which are related to substance metabolism for metabolic water conversion in arid environments, while the genes such as a disintegrin and metalloprotease domain metallopeptidase domain 21 (*Adam21*), adenylate cyclase activating polypeptide 1 (*Adcyap1*), coiled-coil-helix-coiled-coil-helix domain containing 1 (*Chchd1*), and protocadherin alpha 6 (*Pcdha6*), which were related with stimulus response and transmembrane signal receptors, were contracted. In addition, positive selection genes, such as angiopoietin 1 (*Angpt1*), aquaporin 8 (*Aqp8*), ATPase H^+^ transporting v0 subunit A4 (*Atp6v0a4*), calcium voltage-gated channel subunit alpha1 G (*Cacna1g*), calcitonin receptor (*Calcr*), endothelial PAS domain protein 1 (*Epas1*), acyl-coa wax alcohol acyltransferase 2 (*Awat2*), glutathione peroxidase 7 (*Gpx7*), and aldehyde dehydrogenase 18 family member A1 (*Aldh18a1*), are mainly involved in the regulation of blood pressure, osmotic pressure, and water–electrolyte balance. The *Ano9* and *Csmd3* genes, along with the contracted genes *Chchd1* and *Pcdha6*, and the positively selected genes *Calcr*, *Epas1*, and *Gpx7*, were all enriched in the functional category of G-protein coupled receptor activity (GO:0004930). However, there are no shared genes among expanded, contracted, and positively selected genes, indicating that these genes have undergone distinct selection pressures and evolutionary paths during their evolution.

In the wild, the main food sources of *O. sibirica* are the stems and leaves of green plants (such as some species of the family Cucurbitaceae with high water content), as well as a limited number of insect species (Narisu et al. 2009a, 2009b). These rodents also feed on dry seeds rich in carbohydrates (Schmidt-Nielsen 1964), which could produce higher amounts of metabolic water than those produced from lipid or protein substrates (Schmidt-Nielsen and Schmidt-Nielsen 1952). The pathways such as the tricarboxylic acid cycle, starch and sucrose metabolism, glycerophosphate and glyceride metabolism were significantly enriched in the DEGs of *O. sibirica* (Fig. 8). *Aldh7a1*, can oxidize detoxification, and inhibit intracellular transport (Yang et al. 2019), and can also protect cells and tissues from osmotic stress damage by generating osmotic substances, thus participating in processes related to water conversion and affecting the balance of intracellular and extracellular water (Brocker et al. 2010). *Awat2*, encodes diacylglycerol acyltransferase and is involved in energy storage and construction of skin waterproof barrier. A study in mice (*Mus musculus*) showed that the absence of *Awat2* led to a reduction in ceramide and a defect in skin waterproofing (Yen et al. 2005). The increased expression of *Aldh7a1* and *Awat2* in *O. sibirica* from arid habitats or under water deprivation conditions may be involved in the regulation of lipid metabolism and water conversion. Therefore, the enriched fat and carbohydrate metabolic pathways may contribute to the production of metabolic water in jerboas from arid habitats.

The concentration of urea and electrolytes in the urine of *O. sibirica* is increased under arid conditions, and *O. sibirica* has a high tolerance to dehydration under high temperatures (Prakash and Ghosh 2012). We observed that *O. sibirica* can survive for 11 days under the condition of complete water deprivation, indicating that it has strong tolerance to aridity. However, the kidneys of *O. sibirica* have a weaker ability to concentrate urine as evidenced by its lower urine osmotic pressure, which was different from the responses in other desert rodents with concentrated urine under water-deprivation conditions. The enrichment in AA metabolism, the metabolism of xenobiotics by cytochrome P450, the PPAR signaling pathway, and α1 adrenergic receptor activity may be involved in the vascular smooth muscle contraction and promote water reabsorption in the kidneys, whereas downregulated expression of *Lnpep* and *Wnk4*, which are involved in the renin–angiotensin osmotic regulation pathway, in *O. sibirica* in arid environments may reduce angiotensin synthesis, inhibit excessive vascular contraction and maintain osmotic homeostasis and blood pressure.

In addition, *O. sibirica* has unique amino acid substitutions in *Umod* (E444K, N455S) and *Scnn1a* (Y499F, C508L). Both *Umod* and *Scnn1a* play important roles in kidney function and blood pressure regulation. *Scnn1a* encodes the α subunit of the epithelial sodium channel (α-*ENaC*), and *α-ENaC* levels are reduced in response to high sodium intake to excrete excess Na^+^ in the kidney of *M. unguiculatus* (Nouri et al. 2020, 2022). A glycosylated protein-uromodulin encoded by *Umod* is the most abundant urinary protein in mammalian pro-urine under physiological conditions, and is believed to play a role in the transport of sodium, potassium, and calcium within the Henle's loop to maintain water–electrolyte homeostasis (Trudu et al. 2013; Elizabeth et al. 2021). Amino acid substitutions can alter protein function; for example, the P1172T mutation in the period circadian regulator 2 (*PER2*) sequence has been associated with circadian arrhythmia in the *Rangifer tarandus* (Lin et al. 2019). Moreover, a previous study revealed that the genes receptor tyrosine kinase like orphan receptor 2 (*Ror2*) and e2f transcription factor 7 (*E2f7*), involved in kidney development and osmoregulation in the Siberian jerboa, have specific CNE mutations (Chai et al. 2024), and *Ror2* mutant mice exhibit renal hypoplasia and ectopic ureteral bud formation (Kirat et al. 2020 ). These specific variations in coding and noncoding sequences related to the urinary osmoregulation system may be one of the key mechanisms for arid adaptation in the Siberian jerboa.

Aquaporins play a direct role in mediating the process of water reabsorption (Fenton and Knepper 2007; Dantzler et al. 2011; Esteva-Font et al. 2012 ; Nawata and Pannabecker 2018; Alvira-Iraizoz et al. 2021), and the increase in water reabsorption may be a convergent phenomenon in mammals to cope with dehydration. However, this convergent adaptation can vary in wild populations, with differences in key genes associated with the same pathway in different species (Benlamlih et al. 1992; Goumi et al. 1993; Wang et al. 2014; Wu et al. 2014). During water deprivation, *Aqp4* was upregulated, while *Aqp3* was downregulated, accompanied by no changes in *Aqp1* or *Aqp2* in the kidneys of the *O. sibirica*. However, upregulation of *Aqp2* was observed in the kidneys under water restriction conditions for the production of highly concentrated urine in some other small mammals, such as in the *Lepus yarkandensis* (Zhang et al. 2013), *Rattus norvegicus* (Dantzler et al. 2011), *M. unguiculatus* (Xu and Wang 2016), and *M. musculus* (Fenton and Knepper 2007; Esteva-Font et al. 2012), and downregulation of renal *Aqp4* expression associated with reduced urine osmolality was observed in *M. unguiculatus* fed with high salt diets (Yang et al. 2025). The production of highly concentrated urine in the large mammal *C. bactrianus* is achieved by an increase in sodium excretion, and water is effectively stored in the kidney to cope with high-salt and water-deficient environments (Benlamlih et al. 1992; Goumi et al. 1993). The expression of *Aqp1* and *Aqp3* is variably increased in the medulla of the kidney in *Camelus dromedarius* during dehydration (Alvira-Iraizoz et al. 2021). However, *Aqp4* is absent in the camel kidneys (Wang et al. 2014; Wu et al. 2014), while *Aqp3* and *Aqp4* are highly expressed in *O. sibirica*. In addition, the shared DEGs between wild diverse habitats and indoor WS, such as *Cacna1h*, adrenoceptor alpha 1d (*Adra1d*), and *Calhm3*, suggest the critical genes that were involved in processes of osmosis and metabolic regulation for arid adaptation in *O. sibirica*. The low osmotic pressure of urine in *O. sibirica* in arid habitats and its inability to produce concentrated urine under WS may be related to the different expressions of *Aqp1-4* in its kidneys. The exact mechanisms for the coordinated action of *Aqp1-4*, and the function of unique amino acid substitutions in *Umod* and *Scnn1* genes in renal osmoregulation should be further investigated.

In this study, we assembled a high-quality genome of *O. sibirica* and revealed the genetic changes underlying the adaptations of desert mammalian species in arid environments. The positively selected genes such as *Fgf10*, *Fgf11*, *Hoxc4*, *Hoxd1*, and *Bmp4* may be associated with the morphological adaptation with large ears and long limbs for heat dissipation in the desert environment. Pathways associated with AA metabolism, oxidation–reduction reactions, and DNA repair were enriched in both *O. sibirica* and other desert mammals. However, metabolic water production may be a unique adaptation in *O. sibirica*, which differed from other desert rodents that produce highly concentrated urine to conserve water. The convergence in *Lnpep*, *Wnk4*, *C1qc*, *Aldh7a1*, and *Awat2* and specific amino acid replacements in *Umod* and *Scnn1a* may be associated with renal osmoregulation in *O. sibirica*. This study provides a resource for further studies on the genetic underpinnings of desert adaptation mechanisms and has potential implications for the conservation of genetic resources in the context of global climate change. These findings may also provide new insights into the treatment of human kidney stones, salt-sensitive hypertension, and other kidney-related diseases in the future.

## Materials and Methods

### Field Sample Collection

Jerboas were captured between May 2018 and July 2019 from the arid areas of Minle County, Zhangye city, Gansu Province (G6 100°44′56″E, 38°45′14″N, 10 jerboas) and Alxa Left Banner, Alxa League, the Inner Mongolia Autonomous Region (Asi 105°24′15″E, 37°53′14″N, 10 jerboas), and the humid areas of Zhuolu County, Zhangjiakou city, Hebei Province (H4 115°12′17″E, 40°21′42″N, 10 jerboas) and Fengzhen city, Ulanqab city, Inner Mongolia Autonomous Region (M23 113°25′50″E, 40°42′27″N, 10 jerboas). After euthanization with isoflurane, the kidney tissue was removed, quickly frozen in liquid nitrogen and stored in a −80 °C freezer prior to testing.

### Comparative Analysis of the Body Size Characteristics of Rodents

The experiment was conducted in the desert research center of Alxa Left Banner, Alxa League, Inner Mongolia Autonomous Region, in 2022. *Orientallactaga sibirica*, *D. sagitta*, and *M. meridianus* (10 for each species) were captured via live traps, their body weights, tail lengths, ear lengths, and hind leg lengths were measured, and their sex was recorded, along with the sampling time and sampling location.

#### Indoor Water Deprivation Stress Experiment

Twenty *O. sibirica* rodents (10 females and 10 males) were captured from wild habitats and transported to an indoor seminatural environment for a controlled experiment. The animals were provided with standard rat pellet chow (Beijing KeAo Bioscience Co., Beijing, China) and water. After acclimation to the indoor seminatural environment for 2 weeks, the jerboas were divided into two groups: the CK group (provided with food and drinking water *ad libitum*) and the WS group (provided with food *ad libitum* but without drinking water). On the basis of our pilot study under the same conditions, some individuals began to die on the 11th day under water deprivation conditions, so the experiment was carried out for 12 days. At the end of acclimation, the jerboas were euthanized with isoflurane, and blood samples were taken from the orbital venous plexus, incubated at room temperature for 2 h, and centrifuged at 3,000 rpm for 10 min at 4 °C to obtain the serum. The kidneys were collected, quickly frozen in liquid nitrogen, and stored in a −80 °C freezer.

### Comparative Analysis of Urine Osmotic Pressure

In this experiment, we used data from 2 studies conducted by Beuchat in 1990 and 1996 on the maximum urine concentration of mammals, and selected data from 24 rodents for subsequent comparisons in the water restriction stress experiments (Bozinovic and Gallardo 2006; Giorello et al. 2014). The urine osmotic pressure of *O. sibirica* was obtained using 20 rodents who underwent indoor water deprivation in 2021. On day 11 after WS, the jerboas were placed in a metabolic cage, and urine was collected for 24 h. The urine volume, turbidity, and color were recorded. The collected urine was stored in a refrigerator for subsequent comparative analysis. The urine osmotic pressure of *O. sibirica* was measured using an ice point osmometer (SMC-30B, Tianhe Analytical Instruments, Tianjin). A sample of 60 μL was injected into a test tube (0.5 mLl PCR tube), and visible bubbles were removed. The test tube was inserted into the support base to the stop position, allowing the temperature probe to remain fully immersed in the test tube and in the center of the liquid. The test button was clicked, and the handle was moved downward, allowing the temperature probe to be steadily inserted into the cooling pool. When the temperature of the test sample decreased to the cooling temperature (−7 °C), the “result” indicator light turned on, and the resulting data was presented as the osmolarity of the tested sample (mosm kg^−1^).

### Analysis of Blood Biochemical Indicators

Within 1 h after sampling, the blood samples were tested for biochemical indicators via a fully automated animal biochemical testing system (Mindray, BS-240 Vet, Shenzhen) and relevant reagent kits, with a total of five parameters measured, including the A/G, BUN, ALT, Cr, and creatine kinase.

### Animal Ethics

This study was conducted in accordance with the guidelines issued by the Animal Care and Treatment Ethics Committee of Inner Mongolia Agricultural University. The committee requires all researchers and students involved in wildlife and experimental animals to be certified in accordance with the requirements of the Inner Mongolia Agricultural University Ethics Committee (NND2017012 and NND2022093).

### Genome-Wide Sequencing

Muscle tissue was collected from a female Siberian jerboa (*O. sibirica*) captured near the Tengger Desert in the Inner Mongolia Autonomous Region. DNA was isolated using a Qiagen DNA Genomic Kit (Qiagen, Valencia, CA, United States), and the integrity of the DNA was verified using agarose gel electrophoresis. The DNA quantity was assessed via a Qubit fluorometer, and the DNA purity was detected via a Nanodrop spectrophotometer (Thermo Fisher Scientific, Waltham, MA, United States). Four types of whole-genome sequencing data were generated: Illumina short reads, Pacific Biosciences (PacBio) long reads, 10X Genomics reads, and Hi-C reads. For the genome survey, two paired-end libraries with insert sizes of ∼350 bp were constructed according to Illumina's protocol. For the PacBio sequencing, the qualified DNA samples were randomly broken into fragments using a g-TUBE (Covaris), after which the large fragments (≥20 kb) of DNA were enriched and purified with magnetic beads (Myers 2014). DNA fragments were repaired via damage and end repair. Stem ring-like adaptors were added at the two ends of the DNA fragments, and the fragments that failed to be repaired were removed by adding exonuclease. The ∼40-kb SMRTbell libraries were prepared according to the PacBio protocol. We carried out single-molecule real-time (SMRT) sequencing via P6-C4 chemistry on the PacBio Sequel II platform. Two 10X Genomics libraries were prepared using the 10X Genomics Chromium Controller instrument (10X Genomics, Pleasanton, CA, United States) fitted with a microfluidic genome chip according to the Chromium Genome Reagent Kit user guide. After the library was prepared by adding the Illumina P5 and P7 adaptors to the amplification products, PE150 was used for sequencing on the Illumina platform. The chromatin was fixed in the nucleus for Hi-C libraries with formaldehyde and then extracted. Finally, the Hind III restriction enzyme was used to digest the chromatin. The DNA ends were labeled with biotin, and DNA ligation was performed by adding T4 DNA ligase.

### Genome Assembly and Quality Assessment

This study selected the longest single-pass long reads as the seed reads and then mapped these onto the PacBio long reads via Daligner (Myers 2014). We generated a consensus of mapped reads via LASort, LAMerge, and pbdacgon software and then used unitigs of the preassembled reads to generate the layout of overlapping reads and the contigs of the assembly. A high-quality consensus was obtained after the PacBio long reads were mapped to the de novo assembled reference via Quiver (smrtlink 5.0.1) (Chin et al. 2013). The FALCON assembler (RRID:SCR_016089) produced an initial contig with 8,200 primary contigs (Pendleton et al. 2015), after which we improved the accuracy of the assembly via a second round of polishing via Pilon (version 1.22) with Illumina short reads.

The contigs were scaffolded with optical mapping using the 10X genomic sequence. Qualitatively, scaffolds that are physically near each other are present in the same barcoded pools more often than expected by chance. By examining the co-occurrence of reads from the same pool mapped onto the ends of scaffolds, we can identify and orient linked scaffolds. The program fragScaff (Adey et al. 2014) (version 140324) was used to produce an initial scaffold.

Chromosome-scale scaffolding was performed via the Hi-C-based proximity-guided assembly platform based on the LACHESIS method (Burton et al. 2013), which leverages the inverse relationship between genomic distance and proximity contact to group, order, and orient scaffolds into complete chromosomes. The Hi-C PE reads were uniquely mapped onto the PacBio-10X assembly, and then the Hi-C linked signals were used to correct assembly errors, to cluster scaffolds into chromosomal groups, and to order and orient scaffolds on chromosomes.

To assess the completeness of the assembled genome, we performed BUSCO analysis (BUSCO, version 2.0, RRID:SCR_015008) (Simão et al. 2015) by searching against the mammals BUSCOs. The completeness of the genome was assessed via CEGMA (core eukaryotic gene mapping approach) (Parra et al. 2007).

### Genome Annotation

The repetitive sequences were identified in the *O. sibirica* genome via a combination of sequence homology searching and ab initio prediction. Repeat Masker and Repeat Protein Mask were used to search against Repbase for homology-based prediction. Tandem Repeats Finder (TRF), LTR_FINDER, PILER, and Repeat Scout with default parameters were used for *ab initio* prediction. Genes were predicted through a combination of homology-based prediction, *ab initio* prediction, and transcriptome-based prediction methods. BLAST hits were aligned via Solar software 46, and GeneWise (version 2.4.1) was used to predict the exact protein sequence to the corresponding gene sequence of each hit. The homology-based predictions were denoted as “Homology-set.” The RNA-seq data were derived from eye, heart, kidney, liver, lung, muscle, and spleen tissues and then assembled via Trinity (version 2.0) (Grabherr et al. 2011). These assembled sequences were aligned against the *O. sibirica* genome via Program to Assemble Spliced Alignment (PASA), and valid transcript alignments were clustered on the basis of genome mapping location and assembled into gene structures. The gene models created via PASA were denoted as the PASA-T-set (PASA Trinity set). The RNA-seq reads were directly mapped to the genome via TopHat (version 2.0.8) to identify putative exon regions and splice junctions, from which we assembled the gene models via Cufflinks (version 2.1.1) (Cufflinks-set). To predict coding regions in the repeat-masked genome, we used Augustus (RRID:SCR_008417), GeneID, GeneScan, GlimmerHMM (RRID:SCR_002654), and SNAP. We trained Augustus, SNAP, and GlimmerHMM using the PASA-H-set gene models and integrated the gene models from all the methods using Evidence Modeler (EVM). Weights were set for each type of evidence as follows: PASA-T set > Homology set > Cufflinks set > Augustus > GeneID = SNAP = GlimmerHMM = GeneScan. PASA2 was used for updating the gene models and generating UTRs and alternative splicing variation information. The functions of the predicted protein-coding genes were annotated by searching for functional motifs, domains, and possible biological processes of genes in public databases, including SwissProt, Pfam, the NCBI nonredundant (NR), Gene Ontology (GO), and the Kyoto Encyclopedia of Genes and Genomes (KEGG) database.

### Gene Family Cluster Comparative Analysis

The phylogeny, nucleotide and protein data of *O. sibirica* were compared to those of *O. sibirica* and 27 other rodents (*O. cuniculus*, *Ochotona princeps*, *M. musculus*, *Mus caroli*, *R. norvegicus*, *M. unguiculatus*, *M. meridianus*, *Neotoma lepida*, *P. maniculatus*, *Microtus ochrogaster*, *Cricetulus griseus*, *Mesocricetus auratus*, *Nannospalax galili*, *J. jaculus*, *D. sagitta*, *D. ordii*, *Castor canadensis*, *Marmota marmota*, *Ictidomys tridecemlineatus*, *Urocitellus parryii*, *Chinchilla lanigera*, *Octodon degus*, *Cavia porcellus*, *Heterocephalus glaber*, *Fukomys damarensis*), and *Monodelphis domestica* (which belongs to Didelphimorphia, as an outgroup species). All these data except for *O. sibirica* were retrieved from public databases.

The longest transcript was selected from alternative splicing transcripts from one gene; genes with ≤ 50 amino acids were removed. The similarities among filtered protein sequences in these species were identified via all-against-all BLASTP (RRID:SCR_001010) with an *E*-value cut-off of 1E-07. OrthoMCL (RRID: SCR_007839) clustered the genes of these species into gene families with the parameter “-inflation 1.5” (Altschul et al. 1990).

### Phylogenetic Tree Construction and Divergence Time Estimation

A phylogenetic tree was constructed to identify the evolutionary relationships of *O. sibirica* with other 27 species using the nucleic acid sequences for shared single-copy orthologous genes. In brief, the sequences of protein-coding genes were first aligned via the MUSCLE (RRID:SCR_011812) tool with default parameters, which were further concatenated to one supergene sequence for each species to form a data matrix. The phylogenetic tree was constructed via a maximum-likelihood algorithm in RAxML (RRID:SCR_006086), with the optimal amino acid substitution model selected by the PROTGAMMAAUTO parameter (Edgar 2004). The MCMCTree program of PAML (v4.9) was applied to estimate the divergence time with the primary parameters (burn-in = 10,000, sample number = 100,000, and sample frequency = 2). To estimate the divergence time, we used fossil-based calibration via the MCMCTree program in the PAML package (http://www.timetree.org) for *M. musculus* and *M. unguiculatus* (23.1 to 34) (Chevret and Dobigny 2005; Montgelard and Matthee 2012), *F. damarensis* and *H. glaber* (28 to 40) (Winkler 2002; Huchon et al. 2007), *O. cuniculus* and *O. princeps* (48 to 55) (Jacobs and Downs 1994), *M. musculus* and *J. jaculus* (50 to 59) (Li et al. 2005; Pisano et al. 2015), *C. canadensis* and *D. ordii* (56 to 71) (Li et al. 2005; Wu et al. 2012), *M. musculus* and *I. tridecemlineatus* (66 to 75) (Hasegawa et al. 2003; Jacobs and Flynn 2005), *M. musculus* and *C. porcellus* (67 to 79) (Benton et al. 2009; Jardine et al. 2012), *M. domestica* and *M. musculus* (150 to 167) (Archibald et al. 2001; Hasegawa et al. 2003; Beck 2008).

### Expansion and Contraction of Gene Families

The CAFÉ tool (version 4.0) was used to analyze the expansion and contraction of orthologous gene families between each of the 28 species and a common ancestor via a stochastic birth–death model with the lambda parameter (Han et al. 2013). This model was used to examine the changes in gene families along each lineage on the phylogenetic tree. A probabilistic graphical model was subsequently used to calculate the probability of transitions in gene family size from parent nodes to offspring nodes, and *P* values were subsequently calculated for each lineage on the basis of the conditional likelihood.

### Positively Selected Genes

The protein alignments of single-copy gene families were generated in *O. sibirica*, *J. jaculus*, *P. maniculatus*, *M. unguiculatus*, *D. ordii*, and *O. cuniculus* via MUSCLE (RRID:SCR_011812) (Edgar 2004). Gblocks (RRID:SCR_015945) were used to filter poorly aligned positions and divergent regions of the protein alignments before transforming them to coding DNA sequence alignments (Castresana 2000). We conducted comparative genomic analysis using single-copy genes and used *O. sibirica* as the foreground and other species as the background to examine the evolutionary relationships of genes and select positively selected genes. We represented the evolutionary tree of genes using the species evolutionary tree and chose the Branchsite model for analysis. PAML (https://github.com/HLTCHKUST/PAML) was used to detect single-copy orthologs that underwent significant positive selection. The Branchsite model underwent two rounds of analysis. The first control file is null.ctl, with fix_omega = 1 and omega = 1, and the second control file is alte.ctl, with fix_omega = 0 and omega = 1.5. (Zhang et al. 2005). Other detailed parameter files have been uploaded to the website (github.com/jyl0829/Orientallactaga-sibirica-).

### Convergent Evolution Analysis

Genomic data downloaded from public databases were used to detect convergent signatures at the genome-wide level among the rodent species adapted to desert environments (rodents in arid habitats, *O. sibirica*, *J. jaculus*, *M. unguiculatus*, and *D. ordii*; and rodents in moist habitats, *P. maniculatus* and *O. cuniculus*). The single-copy gene sets were identified via OrthoFinder (version 2.2.7) (Emms and Kelly 2015), PRANK (version 170427), and trimAl (version 1.4) software to align and cull unaligned sequences (Capella-Gutiérrez et al. 2009). Orthologs shorter than 50 amino acids were removed, and ultimately, a total of 7,999 orthologous genes were obtained for reconstructing ancestral protein sequences. We used the Codeml program in PAML 4.7 to reconstruct ancestral protein sequences through 7,999 orthologs among six species with parameters (RateAncestor = 1, model = 2). To infer ancestral status, we simulated amino acid status on the basis of amino acid frequency and tree topology using the JTT + gamma amino acid substitution model and EVOLVE in PAML. The characters with the highest posterior probabilities were considered ancestral. Finally, we estimated the accuracy of the ancestral status by comparing the status of the outgroup to the simulated status (Xu et al. 2017).

### Comparative RNA Sequencing Analysis of the Siberian Jerboa Kidney Tissues

The RNA from the kidney tissues was sequenced, and three biological replicates were established for DEG analysis. After quality inspection, a chain-specific library was constructed via ribosomal RNA removal. Prior to DEG analysis, the read counts of each sequencing library were adjusted via a proportional normalization factor via the edge R package. Samples were subjected to DEG analysis via the edge R package (3.12.1), and *P* value adjustments were performed via the Benjamini & Hochberg method. DEG significance was assessed as a corrected *P* value < 0.05 and |log2folodchange| > 1.

### Real-Time Quantitative PCR

The RT-qPCR experiment was performed as follows: the cDNA samples (1 μL) were utilized as a template for the following PCR reaction applying gene-specific primers (Table S31). Using the reverse transcription kit (TUREscript 1st Stand cDNA SYNTHESIS Kit, PC1802 from Adlai) and the fluorescent reagent kit (2×SYBR^®^ Green MIX, PC3302, Adlai), we performed reverse transcription operations. The final total reaction volume of 20 μL contained 5 μL of 2×SYBR^®^ Green Supermix, 0.5 μL of forward primer, 0.5 μL reverse primer, and 3 μL RNase-free ddH2O. After the first polymerase activation step at 95 °C for 3 min, amplification was carried out by 39 cycles (95 °C for 10 s and 60 °C for 30 s + plate read). The built-in melting curve was completed after the process (60 to 95°C + 1°C/cycle, holding time 4 s). Using Gapdh expression as an internal reference, the relative amount of gene expression in each sample (repeat count = 3) was calculated using 2^−ΔΔCT^ methods (Schmittgen and Livak 2008).

### Statistical Analysis

The body size data were calibrated on the basis of body weight before interspecies differences were analyzed. IBM SPSS Statistics 24 was used to perform a one-way ANOVA on the body size data of three rodent species captured in Alxa, as well as the jerboas captured from various wild distribution areas. IBM SPSS Statistics 24 was used to analyze the difference in the maximum urine osmotic pressure and the blood biochemical indicators of various rodent species. The AI is equal to the ratio of potential evapotranspiration (PE) to precipitation (P), calculated as follows: AI = PE/P.

Heatmaps and enrichment maps were created via an online platform dedicated to data analysis and visualization (https://www.bioinformatics.com.cn), whereas bar charts and violin plots were generated by Origin 2021, and entry plots were generated by Adobe Illustrator 2023.

## Supplementary Material

msaf230_Supplementary_Data

## Data Availability

All genome-sequencing data generated in this project are available at NCBI BioProject under accession code PRJNA1181308.

## References

[msaf230-B1] Ababaikeri B et al Whole-genome sequencing of Tarim red deer (*Cervus elaphus yarkandensis*) reveals demographic history and adaptations to an arid-desert environment. Front Zool. 2020:17:31. 10.1186/s12983-020-00379-5. 33072165 PMC7565370

[msaf230-B2] Adey A et al In vitro, long-range sequence information for de novo genome assembly via transposase contiguity. Genome Res. 2014:24:2041–2049. 10.1101/gr.178319.114. 25327137 PMC4248320

[msaf230-B3] Allen JA . The influence of physical conditions in the genesis of species. Radic Rev. 1877:1:108–140. 10.1038/scientificamerican05251907-26247supp.

[msaf230-B4] Altschul SF, Gish W, Miller W, Myers EW, Lipman DJ. Basic local alignment search tool. J Mol Biol. 1990:215:403–410. 10.1016/S0022-2836(05)80360-2. 2231712

[msaf230-B5] Alvira-Iraizoz F et al Multiomic analysis of the Arabian camel (*Camelus dromedarius*) kidney reveals a role for cholesterol in water conservation. Commun Biol. 2021:4:779. 10.1038/s42003-021-02327-3. 34163009 PMC8222267

[msaf230-B6] Archibald JD, Averianov AO, Ekdale EG. Late Cretaceous relatives of rabbits, rodents, and other extant eutherian mammals. Nature. 2001:414:62–65. 10.1038/35102048. 11689942

[msaf230-B7] Beck RM . A dated phylogeny of marsupials using a molecular supermatrix and multiple fossil constraints. J Mammal. 2008:89:175–189. 10.1644/06-MAMM-A-437.1.

[msaf230-B8] Benlamlih S, Dahlborn K, Filali RZ, Hossaini-Hilali J. Fluid retention after oral loading with water or saline in camels. Am J Physiol Regul Integr Comp Physiol. 1992:262:R915–R920. 10.1152/ajpregu.1992.262.5.R915. 1590486

[msaf230-B9] Benton MJ, Donoghue PC, Asher RJ. Calibrating and constraining molecular clocks. J Vertebr Paleontol. 2009:29:35–86. 10.1093/oso/9780199535033.003.0004.

[msaf230-B10] Bozinovic F, Gallardo P. The water economy of South American desert rodents: from integrative to molecular physiological ecology. Comp Biochem Physiol C Toxicol Pharmacol. 2006:142:163–172. 10.1016/J.CBPC.2005.08.004. 16198637

[msaf230-B11] Brocker C et al Aldehyde dehydrogenase 7A1 (*ALDH7A1*) is a novel enzyme involved in cellular defense against hyperosmotic stress. J Biol Chem. 2010:285:18452–18463. 10.1074/jbc.M109.077925. 20207735 PMC2881771

[msaf230-B12] Burton JN et al Chromosome-scale scaffolding of de novo genome assemblies based on chromatin interactions. Nat Biotechnol. 2013:31:1119–1125. 10.1038/nbt.2727. 24185095 PMC4117202

[msaf230-B13] Capella-Gutiérrez S, Silla-Martínez JM, Gabaldón T. Trimal: a tool for automated alignment trimming in large-scale phylogenetic analyses. Bioinformatics. 2009:25:1972–1973. 10.1093/bioinformatics/btp348. 19505945 PMC2712344

[msaf230-B14] Castresana J . Selection of conserved blocks from multiple alignments for their use in phylogenetic analysis. Mol Biol Evol. 2000:17:540–552. 10.1093/oxfordjournals.molbev.a026334. 10742046

[msaf230-B15] Chai S et al Genomic insights into adaptation to bipedal saltation and desert-like habitats of jerboas. Sci China Life Sci. 2024:67:2003–2015. 10.1007/s11427-023-2516-9. 38902451

[msaf230-B16] Cheng JL et al Phylogeny and taxonomic reassessment of jerboa, *Dipus* (Rodentia, Dipodinae), in inland Asia. Zool Scr. 2018:47:630–644. 10.1111/zsc.12303.

[msaf230-B17] Cheviron ZA, Bachman GC, Connaty AD, McClelland GB, Storz JF. Regulatory changes contribute to the adaptive enhancement of thermogenic capacity in high-altitude deer mice. Proc Natl Acad Sci U S A. 2012:109:8635–8640. 10.1073/pnas.1120523109. 22586089 PMC3365185

[msaf230-B18] Chevret P, Dobigny G. Systematics and evolution of the subfamily Gerbillinae (Mammalia, Rodentia, Muridae). Mol Phylogenet Evol. 2005:35:674–688. 10.1016/j.ympev.2005.01.001. 15878135

[msaf230-B19] Chin C-S et al Nonhybrid, finished microbial genome assemblies from long-read SMRT sequencing data. Nat Methods. 2013:10:563–569. 10.1038/nmeth.2474. 23644548

[msaf230-B20] Cooper KL . The lesser Egyptian jerboa, *Jaculus jaculus*: a unique rodent model for evolution and development. Cold Spring Harb Protoc. 2011:2011:pdb. emo066704. 10.1101/pdb.emo066704. 22135653

[msaf230-B21] Dantzler W, Pannabecker T, Layton A. Urine concentrating mechanism in the inner medulla of the mammalian kidney: role of three-dimensional architecture. Acta Physiol. 2011:202:361–378. 10.1111/j.1748-1716.2010.02214.x. PMC3807677 21054810

[msaf230-B22] Edgar RC . MUSCLE: multiple sequence alignment with high accuracy and high throughput. Nucleic Acids Res. 2004:32:1792–1797. 10.1093/nar/gkh340. 15034147 PMC390337

[msaf230-B23] Elizabeth VFL et al Effect of a common *UMOD* variant on kidney function, blood pressure, cognitive and physical function in a community-based cohort of older adults. J Hum Hypertens. 2021:36:983–988. 10.1038/s41371-021-00608-2. 34593962 PMC9649423

[msaf230-B24] Emms DM, Kelly S. OrthoFinder: solving fundamental biases in whole genome comparisons dramatically improves orthogroup inference accuracy. Genome Biol. 2015:16:157. 10.1186/s13059-015-0721-2. 26243257 PMC4531804

[msaf230-B25] Esteva-Font C, Ballarin J, Fernández-Llama P. Molecular biology of water and salt regulation in the kidney. Cell Mol Life Sci. 2012:69:683–695. 10.1007/s00018-011-0858-4. 21997386 PMC11114984

[msaf230-B26] Evans AR et al The maximum rate of mammal evolution. Proc Natl Acad Sci U S A. 2012:109:4187–4190. 10.1073/pnas.1120774109. 22308461 PMC3306709

[msaf230-B27] Fenton RA, Knepper MA. Mouse models and the urinary concentrating mechanism in the new millennium. Physiol Rev. 2007:87:1083–1112. 10.1152/physrev.00053.2006. 17928581

[msaf230-B28] Giorello FM et al Characterization of the kidney transcriptome of the South American olive mouse *Abrothrix olivacea*. BMC Genomics. 2014:15:1–10. 10.1186/1471-2164-15-446. 24909751 PMC4189146

[msaf230-B29] Goumi MB et al Hormonal control of water and sodium in plasma and urine of camels during dehydration and rehydration. Gen Comp Endocrinol. 1993:89:378–386. 10.1006/gcen.1993.1045. 8335227

[msaf230-B30] Grabherr MG et al Full-length transcriptome assembly from RNA-Seq data without a reference genome. Nat Biotechnol. 2011:29:644–652. 10.1038/nbt.1883. 21572440 PMC3571712

[msaf230-B31] Han MV, Thomas GW, Lugo-Martinez J, Hahn MW. Estimating gene gain and loss rates in the presence of error in genome assembly and annotation using CAFE 3. Mol Biol Evol. 2013:30:1987–1997. 10.1093/molbev/mst100. 23709260

[msaf230-B32] Hasegawa M, Thorne JL, Kishino H. Time scale of eutherian evolution estimated without assuming a constant rate of molecular evolution. Genes Genet Syst. 2003:78:267–283. 10.1266/ggs.78.267. 14532706

[msaf230-B33] Haugen TO, Vøllestad LA. Population differences in early life-history traits in grayling. J Evol Biol. 2010:13:897–905. 10.1046/j.1420-9101.2000.00242.x.

[msaf230-B34] Hill RW, Veghte JH. Jackrabbit ears: surface temperatures and vascular responses. Science. 1976:194:436–438. 10.1126/science.982027. 982027

[msaf230-B35] Hu CK, Hoekstra HE. Peromyscus burrowing: a model system for behavioral evolution. Sem Cell Develop Biol. 2017:107–114. 10.1016/j.semcdb.2016.08.001. 27496333

[msaf230-B36] Huchon D, et al Multiple molecular evidences for a living mammalian fossil. Proc Natl Acad Sci U S A. 2007:104:7495–7499. 10.1073/pnas.0701289104. 17452635 PMC1863447

[msaf230-B37] Hyndman KA, Pannabecker TL. Osmoregulation in desert-adapted mammals. Springer; 2015. 10.1007/978-1-4939-3213-9:191-211. 10.1007/978-1-4939-3213-9_10.

[msaf230-B38] Ivy CM, Scott GR. Control of breathing and ventilatory acclimatization to hypoxia in deer mice native to high altitudes. Acta Physiol. 2017:221:266–282. 10.1111/apha.12912. 28640969

[msaf230-B39] Jacobs LL, Downs WR. The evolution of murine rodents in Asia. In: Tomida Y, Li CK, Setoguschi T, editors. Rodent and lagomorph families of Asian origins and their diversification. National Science Museum Monograph; 1994. p. 149–156. ISSN:13429574.

[msaf230-B40] Jacobs LL, Flynn LJ. Of mice… again: the Siwalik rodent record, murine distribution, and molecular clocks. In: Lieberman DE, Smith RJ, Kelley J, editors. Interpreting the past: essays on human, primate, and mammal evolution. Brill Academic Publishers; 2005. p. 63–80. 10.1163/9789047416616_011.

[msaf230-B41] James FC . Geographic size variation in birds and its relationship to climate. Ecology. 1970:51:365–390. 10.2307/1935374.

[msaf230-B42] Jardine PE, Janis CM, Sahney S, Benton MJ. Grit not grass: concordant patterns of early origin of hypsodonty in *Great Plains* ungulates and glires. Palaeogeogr Palaeoclimatol. 2012:365–366:1–10. 10.1016/j.palaeo.2012.09.001.

[msaf230-B43] Jiang Z . Mammal diversity and geographical distribution in China. Science Press; 2015.

[msaf230-B44] Kirat E, Albayrak HM, Sahinoglu B, Gurler AI, Karaer K. Autosomal recessive Robinow syndrome with novel ROR2 variants: distinct cases exhibiting the clinical variability. Clin Dysmorphol. 2020:29:137–140. 10.1097/MCD.0000000000000319. 32195677

[msaf230-B45] Kusano E, Tian S, Umino T. Arginine vasopressin inhibits interleukin-1 beta-stimulated nitric oxide and cyclic guanosine monophosphate production via the V1 receptor in cultured rat vascular smooth muscle cells. J Hypertens. 1997:15:627–632. 10.1097/00004872-199715050-00017. 9218182

[msaf230-B46] Li Q, Zheng SH. Note on four species of *dipodids* (Dipodidae, Rodentia) from the Late Miocene Bahe Formation. Lantian, Shaanxi. Vert Palasiat. 2005:43:283–296. https://www.vertpala.ac.cn/EN/Y2005/V43/I04/283.

[msaf230-B47] Lin Z et al Biological adaptations in the Arctic cervid, the reindeer (*Rangifer tarandus*). Science. 2019:364:eaav6312. 10.1126/science.aav6312. 31221829

[msaf230-B48] Mitchell D, Snelling EP, Hetem RS. Revisiting concepts of thermal physiology: predicting responses of mammals to climate change. J Anim Ecol. 2018:87:956–973. 10.1111/1365-2656.12704. 29479693

[msaf230-B49] Monarca RI, Speakman JR, Mathias M. Energetics and thermal adaptation in semifossorial pine-voles *Microtus lusitanicus* and *Microtus duodecimcostatus*. J Comp Physiol B. 2019:189:309–318. 10.1007/s00360-018-1206-1. 30719532

[msaf230-B50] Montgelard C, Matthee CA. Tempo of genetic diversification in southern African rodents: the role of Plio-Pleistocene climatic oscillations as drivers for speciation. Acta Oecol. 2012:42:50–57. 10.1016/j.actao.2012.02.001.

[msaf230-B51] Myers G . Efficient local alignment discovery amongst noisy long reads. Springer; 2014.

[msaf230-B52] Narisu , Suhe, Wu XD. The influence of different grazing systems on the diet habit of *Allactaga sibirica*. Chin J Grassland. 2009b:31:116–120. https://kns.cnki.net/CNKI:SUN:ZGCD.0.2009-04-022.

[msaf230-B53] Narisu N, Suhe S, Wu XD. Botanic food preference of *Allactaga sibirica* Forster and its relationship with the vegetation conditions of their habitat. Acta Agrestia Sin. 2009a:17:383–388. 10.11733/j.issn.1007-0435.2009.03.022.

[msaf230-B54] Nawata CM, Pannabecker TL. Mammalian urine concentration: a review of renal medullary architecture and membrane transporters. J Comp Physiol B. 2018:188:899–918. 10.1007/s00360-018-1164-3. 29797052 PMC6186196

[msaf230-B55] Nouri Z, Zhang XY, Khakisahneh S, Degen AA, Wang DH. The microbiota-gut-kidney axis mediates host osmoregulation in a small desert mammal. NPJ Biofilms Microbiomes. 2022:8:16. 10.1038/s41522-022-00280-5. 35379849 PMC8980004

[msaf230-B56] Nouri Z, Zhang XY, Wang DH. The physiological and molecular mechanisms to maintain water and salt homeostasis in response to high salt intake in Mongolian gerbils (*Meriones unguiculatus*). J Comp Physiol B. 2020:190:641–654. 10.1007/s00360-020-01287-0. 32556536

[msaf230-B57] Parra G, Bradnam K, Korf I. CEGMA: a pipeline to accurately annotate core genes in eukaryotic genomes. Bioinformatics. 2007:23:1061–1067. 10.1093/bioinformatics/btm071. 17332020

[msaf230-B58] Pendleton M et al Assembly and diploid architecture of an individual human genome via single-molecule technologies. Nat Methods. 2015:12:780–786. 10.1038/nmeth.3454. 26121404 PMC4646949

[msaf230-B59] Pisano J et al Out of Himalaya: the impact of past Asian environmental changes on the evolutionary and biogeographical history of Dipodoidea (Rodentia). J Biogeogr. 2015:42:856–870. 10.1111/jbi.12476.

[msaf230-B60] Prakash I, Ghosh PK. Rodents in desert environments. Springer Science & Business Media; 2012.

[msaf230-B61] Qiu Q et al . The yak genome and adaptation to life at high altitude. Nat Genet. 2012:44:946–949. 10.1038/ng.2343. 22751099

[msaf230-B62] Rocha JL, Brito JC, Nielsen R, Godinho R. Convergent evolution of increased urine-concentrating ability in desert mammals. Mamm Rev. 2021b:51:482–491. 10.1111/mam.12244.

[msaf230-B63] Rocha JL, Godinho R, Brito JC, Nielsen R. Life in deserts: the genetic basis of mammalian desert adaptation. Trends Ecol Evol. 2021a:36:637–650. 10.1016/j.tree.2021.03.007. 33863602 PMC12090818

[msaf230-B64] Ryding S, Klaassen M, Tattersall GJ, Gardner JL, Symonds MRE. Shape-shifting: changing animal morphologies as a response to climatic warming. Trends Ecol Evol. 2021:36:1036–1048. 10.1016/j.tree.2021.07.006. 34507845

[msaf230-B65] Schmidt-Nielsen K . Desert animals: physiological problems of heat and water. Biol Med Sci. 1964:144:715–716. 10.1126/science.144.3619.715. 17807044

[msaf230-B66] Schmidt-Nielsen K, Schmidt-Nielsen B. Water metabolism of desert mammals. Physiol Rev. 1952:32:135–166. 10.1152/physrev.1952.32.2.135. 14929697

[msaf230-B67] Schmittgen T, Livak K. Analyzing real-time PCR data by the comparative CT method. Nat Protoc. 2008:3:1101–1108. 10.1038/nprot.2008.73. 18546601

[msaf230-B68] Sears K, Maier JA, Sadier A, Sorensen D, Urban DJ. Timing the developmental origins of mammalian limb diversity. Genesis. 2018:56:e23079. 10.1002/dvg.23079. 29095555

[msaf230-B69] Sears KE . Molecular determinants of bat wing development. Cells Tissues Organs. 2008:187:6–12. 10.1159/000109959. 18160799

[msaf230-B70] Sequencing TBCG, Consortium A. Genome sequences of wild and domestic bactrian camels. Nat Commun. 2012:3:1202. 10.1038/ncomms3089. 23149746 PMC3514880

[msaf230-B71] Simão FA, Waterhouse RM, Ioannidis P, Kriventseva EV, Zdobnov EM. BUSCO: assessing genome assembly and annotation completeness with single-copy orthologs. Bioinformatics. 2015:31:3210–3212. 10.1093/bioinformatics/btv351. 26059717

[msaf230-B72] Storz JF, Cheviron ZA, McClelland GB, Scott GR. Evolution of physiological performance capacities and environmental adaptation: insights from high-elevation deer mice (*Peromyscus maniculatus*). J Mammal. 2019:100:910–922. 10.1093/jmammal/gyy173. 31138949 PMC6533030

[msaf230-B73] Tigano A, Colella JP, MacManes MD. Comparative and population genomics approaches reveal the basis of adaptation to deserts in a small rodent. Mol Ecol. 2020:29:1300–1314. 10.1111/mec.15401. 32130752 PMC7204510

[msaf230-B74] Trudu M et al Common noncoding *UMOD* gene variants induce salt-sensitive hypertension and kidney damage by increasing uromodulin expression. Nat Med. 2013:19:1655–1660. 10.1038/nm.3384. 24185693 PMC3856354

[msaf230-B75] Tseng M et al Decreases in beetle body size linked to climate change and warming temperatures. J Anim Ecol. 2018:87:647–659. 10.1111/1365-2656.12789. 29380382

[msaf230-B76] Walsberg GE . Small mammals in hot deserts: some generalizations revisited. BioScience. 2000:50:109–120. 10.1641/0006-3568(2000)050.

[msaf230-B77] Wang J, Li H, Huang Z, Shao B, Wang J. Renal expression and functions of *AQP1* and *AQP2* in bactrian camel (*Camelus bactrianus*). J Camel Pract Res. 2014:21:153–160. 10.5958/2277-8934.2014.00027.7.

[msaf230-B78] Weeks BC et al Shared morphological consequences of global warming in North American migratory birds. Ecol Lett. 2020:23:316–325. 10.1111/ele.13434. 31800170

[msaf230-B79] Williams JB, Muñoz-Garcia A, Ostrowski S, Tieleman BI. A phylogenetic analysis of basal metabolism, total evaporative water loss, and life-history among foxes from desert and mesic regions. J Comp Physiol B. 2004:174:29–39. 10.1007/s00360-003-0386-0. 14564467

[msaf230-B80] Willmer P, Stone G, Johnston I. Environmental physiology of animals. John Wiley & Sons; 2009. ISBN13: 978-1-444-30922-5.

[msaf230-B81] Winkler AJ . Neogene paleobiogeography and East African paleoenvironments: contributions from the Tugen Hills rodents and lagomorphs. J Hum Evol. 2002:42:237–256. 10.1006/jhev.2001.0501. 11795976

[msaf230-B82] Wu HG et al Camelid genomes reveal evolution and adaptation to desert environments. Nat Commun. 2014:5:5188. 10.1038/ncomms6188. 25333821

[msaf230-B83] Wu SY et al Molecular and paleontological evidence for a post-Cretaceous origin of rodents. PLoS One. 2012:7:e46445. 10.1371/journal.pone.0046445. 23071573 PMC3465340

[msaf230-B84] Xu MM, Wang DH. Water deprivation up-regulates urine osmolality and renal aquaporin 2 in Mongolian gerbils (*Meriones unguiculatus*). Comp Biochem Physiol A Mol Integr Physiol. 2016:194:37–44. 10.1016/j.cbpa.2016.01.015. 26806059

[msaf230-B85] Xu S et al . Genome-wide convergence during evolution of mangroves from woody plants. Mol Biol Evol. 2017:34:1008–1015. 10.1093/molbev/msw277. 28087771

[msaf230-B86] Yang J et al Whole-genome sequencing of native sheep provides insights into rapid adaptations to extreme environments. Mol Biol Evol. 2016:33:2576–2592. 10.1093/molbev/msw129. 27401233 PMC5026255

[msaf230-B87] Yang JS et al *ALDH7A1* inhibits the intracellular transport pathways during hypoxia and starvation to promote cellular energy homeostasis. Nat Commun. 2019:10:4068. 10.1038/s41467-019-11932-0. 31492851 PMC6731274

[msaf230-B88] Yang XZ, Wang CZ, Wang DH, Zhang XY. Specific adaptive mechanisms in water-sodium regulation in a desert rodent fed with salty diets. Compr Physiol. 2025:15:e70015. 10.1002/cph4.70015. 40375467

[msaf230-B89] Yen CLE, Brown IV CH, Monetti M, Farese RV. A human skin multifunctional O-acyltransferase that catalyzes the synthesis of acylglycerols, waxes, and retinyl esters. J Lipid Res. 2005:46:2388–2397. 10.1194/jlr.M500168-JLR200. 16106050 PMC1540095

[msaf230-B90] Zhang J, Nielsen R, Yang Z. Evaluation of an improved branch-site likelihood method for detecting positive selection at the molecular level. Mol Biol Evol. 2005:22:2472–2479. 10.1093/molbev/msi237. 16107592

[msaf230-B91] Zhang JP, Yu WJ. The role of aquaporins in the kidney of Tarim rabbits in adapting to arid environments. Shou Lei Xue Bao. 2013:33:377. 10.16829/j.slxb.2013.04.012.

[msaf230-B92] Zhou X et al Population genomics reveals low genetic diversity and adaptation to hypoxia in snub-nosed monkeys. Mol Biol Evol. 2016:33:2670–2681. 10.1093/molbev/msw150. 27555581

