# Supplementary material for: Multiomic Analyses Reveal the Molecular Mechanisms of Arid Adaptation in a Desert Rodent Species
Source: Mol Biol Evol. 2025 Sep 17;42(10):msaf230. doi: 10.1093/molbev/msaf230 (PMC12502661; doi:10.1093/molbev/msaf230)
Supplement: msaf230_Supplementary_Data [file msaf230_supplementary_data.zip › Supplementary materials.pdf]

Supplementary material

Supplementary Table 1. Sequencing data output

Library	Insert size	Raw reads	Raw base（bp）	Effective rate（%）	Clean base（bp）	Error rate（%）	Q20（%）	Q30（%）	GC content（%）
DES03113-V	350 bp	479,535,004	143,860,501,200	99.54	143,196,626,700	0.03	96.16	91.46	42.04

Supplementary Table 2. Analysis of estimated genomic characteristics

K-mer	K-mer number	K-mer depth	Genome size（Mbp）	Revised genome Size （Mbp）	Heterozygous ratio（%）	Repeat（%）
17	111,916,617,492	34	3,291.67	3,256.43	0.56	65.44

Note: The repeat percentage is based on the k-mer value and represents a mathematically repeated region, which is different from biological replicates (i.e., functional repeating elements).

Supplementary Table 3. Assembly data

	Total length(bp）	Total number	Max length（bp）	N50 length（bp）	N90 length（bp）
contig	2,787,270,534	4,994,513	65,448	1,527	163
scaffold	2,875,817,798	3,906,712	116,399	2,764	218

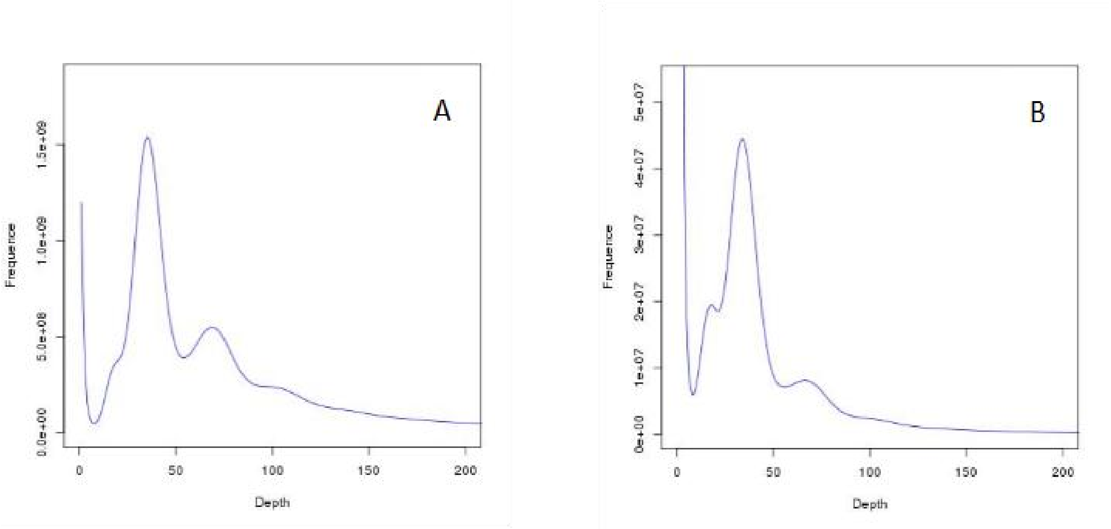

Supplementary Fig. 1 Depth and frequency of K-mer values

Note: The x-axis represents the depth of the kmer (number of kmer occurrences), whereas the y-axis represents the number of kmers at the corresponding depth. The species frequency distribution map is shown in A, and the frequency distribution map is shown in B.

Supplementary Table 4. Genome CEGMA assessment

species	complete		complete + partial	
	# Prots	% completeness	# Prots	% completeness
O. sibirica	226	91.13	241	97.18

Note: “complete” in the Table refers to core gene being >70% assembled; “complete+partial” refers to the core gene being partially assembled.

Supplementary Table 5. Statistics of the genome sequencing data

Pair-end libraries	Insert size	Total data（G）	Read length（bp）	Sequence coverage（X）
Illumina reads	350 bp	286.30	150	87.82
PacBio reads	--	289.83	--	88.90
10X Genomics	600 bp	275.24	150	84.43
Hi-C	350 bp	345.81	150	106.08
Total	-	1197.18	-	367.23

Supplementary Table 6. Genomic statistics of the chromosomes

Sample ID	Length		Number	
	Contig**(bp)	Scaffold (bp)	Contig**	Scaffold
Total	3,154,150,629	3,154,595,829	8,200	3,748
Max	20,928,897	220,870,487	-	-
Number>=2000	-	-	8,073	3,623
N50	3,771,657	153,974,211	227	9
N60	2,663,213	142,180,965	326	11
N70	1,772,004	1,129,053,110	472	14
N80	993,004	91,435,716	711	17
N90	189,000	82,240,493	1,403	20

Note: ** means Contig after scaffolding.

Supplementary Table 7. Genome loading rate

	Number
Mounting base	3,005,174,708
Total base number	3,154,595,829
Mount rate	95.26%

Supplementary Table 8 Chromosome length results.

Chr_Id	Cluster_Number	Chr_Length
chr1	226	220,870,487
chr2	175	197,536,912
chr3	172	195,706,720
chr4	359	189,666,386
chr5	220	188,768,819
chr6	175	184,434,262
chr7	223	175,070,998
chr8	302	163,054,033
chr9	145	163,054,033
chr10	141	142,538,430
chr11	143	142,180,965
chr12	113	116,934,277
chr13	138	114,788,701
chr14	105	112,905,310
chr15	158	110,109,141
chr16	140	104,109,154
chr17	311	91,435,716
chr18	209	91,241,979
chr19	156	85,154,797
chr20	75	82,240,493
chr21	511	52,075,815
chr22	116	46,071,531
chr23	141	38,782,851
chr24	22	4,522,720
Scaffold*	3724	149,421,121

Supplementary Table 9. Repeating sequence statistics

Type	Repeat size（bp）	% of genome
Trf	273,718,051	8.68
Repeat masker	1,595,908,668	50.59
Protein mask	177,099,423	5.61
Total	1,648,830,908	52.27

Note: Total is the nonredundant result obtained by removing the overlapping parts between the above methods.

Supplementary Table 10 Repeated sequence classification results

	Denovo+Repbase Length（bp）	% in Genome	TE proteins length（bp）	% in Genome	Combined TEs length（bp）	% in Genome
DNA	3,129,887	0.1	1,099,048	0.03	3,828,075	0.12
LINE	643,114,020	20.39	138,135,801	4.38	654,833,816	20.76
SINE	252,155,049	7.99	0	0	252,155,049	7.99
LTR	335,472,292	10.63	37,904,715	1.2	346,216,694	10.97
Simple_repeat	1,575,247	0.05	0	0	1,575,247	0.05
Unknown	748,722,464	23.73	0	0	748,722,464	23.73
Total	1,595,908,668	50.59	177,099,423	5.61	1,606,602,448	50.93

Note: Denovo+Repbase represents data from RepeatModeller, Repeat Scout, and LTR. The library predicted by FINDER software was combined with the RepBase nucleic acid library. Ucluster software was used for integration according to the 80-80-80 principle, and then repeat masker software was used to annotate the TEs obtained from the genome. TE proteins represent TEs annotated using the RepBase protein library through Repeat Protein Mask software. Combined TEs represent the integration of the above methods and with redundancies eliminated. Unknown represents repeating sequences that could not be classified by Repeat Mask.

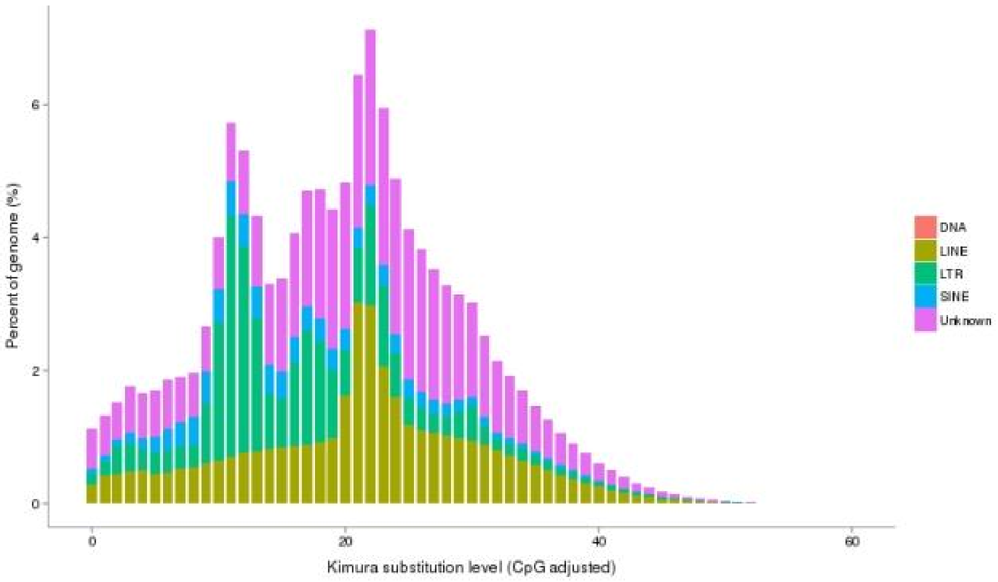

Supplementary Fig. 2 The frequency distribution of transposable element (TE) divergence

Note: The abscissa represents the divergence between the TE sequences annotated in the genome of the Siberian jerboa and the corresponding sequence in Repbase; the vertical axis represents the percentage of TE sequences in the genome under this divergence, with different TEs marked in different colours.

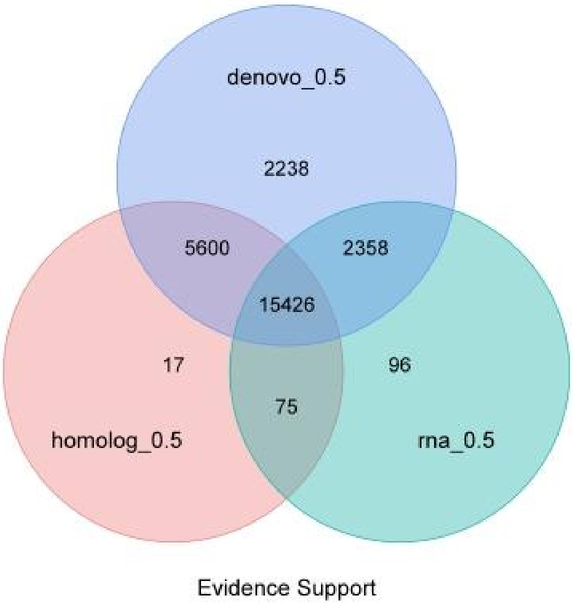

Supplementary Fig. 3 Supporting statistics for the gene analyses

Note: De novo, EVM integrates genes supported by de novo prediction; Homologue, genes supported by homologous prediction during EVM integration; RNA-seq-supported genes during the integration of RNA and EVM; each evidence support is based on the criterion of gene overlap greater than 50%; the numbers represent the number of genes.

Supplementary Table 11 Basic gene sequence characteristics among several sympatric rodent species

Species	Number	Average transcript length （bp）	Average CDS length（bp）	Average exons per gene	Average exon length（bp）	Average intron length（bp）
Orientallactaga sibirica	25,812	33,406.60	1,370.63	8	171.27	4,574.72
Cricetulus griseus	23,687	15,585.85	1,310.94	7.29	179.77	2,268.69
Homo sapiens	22,927	46,242.75	1,710.41	9.65	177.22	5,147.43
Jaculus jaculus	18,849	48,661.75	1,702.84	9.87	172.53	5,294.18
Mus musculus	23,584	37,071.40	1,641.60	9.05	181.3	4,398.74
Peromyscus maniculatu	21,607	38,352.21	1,637.23	9.21	177.83	4,473.91
Rattus norvegicus	22,926	38,361.17	1,611.87	9.04	178.28	4,570.09

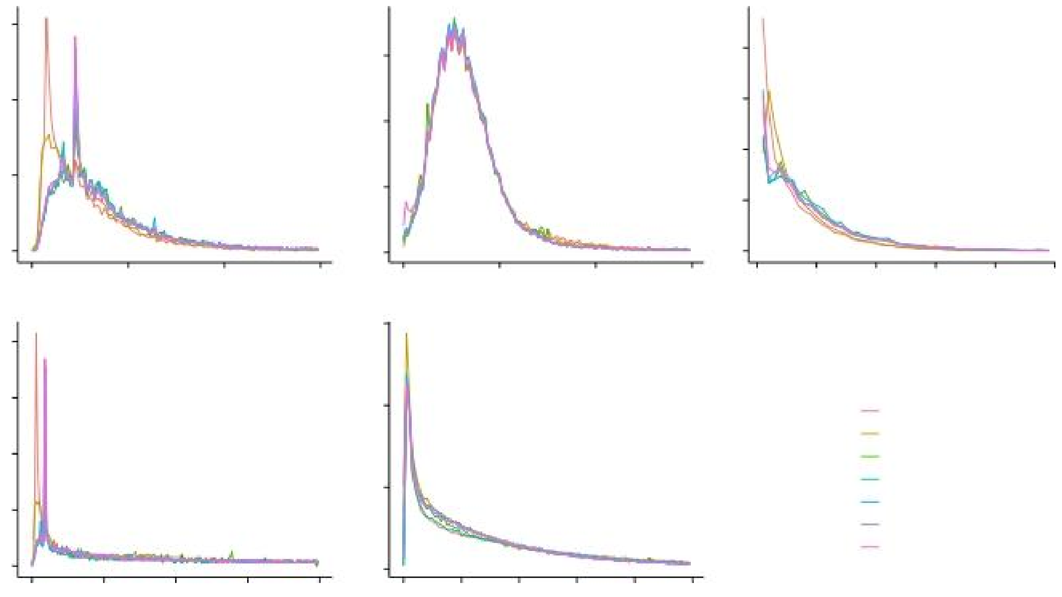

Supplementary Fig. 4 Comparison of sympatric species

Supplementary Table 12 Statistical results of gene function annotation

	Number	Percent（%）
Total	25,812	-
Swissprot	22,861	88.6
Nr	23,368	90.5
KEGG	20,219	78.3
InterPro	25,590	99.1
GO	23,592	91.4
Pfam	19,471	75.4
Annotated	25,661	99.4
Unannotated	151	0.6

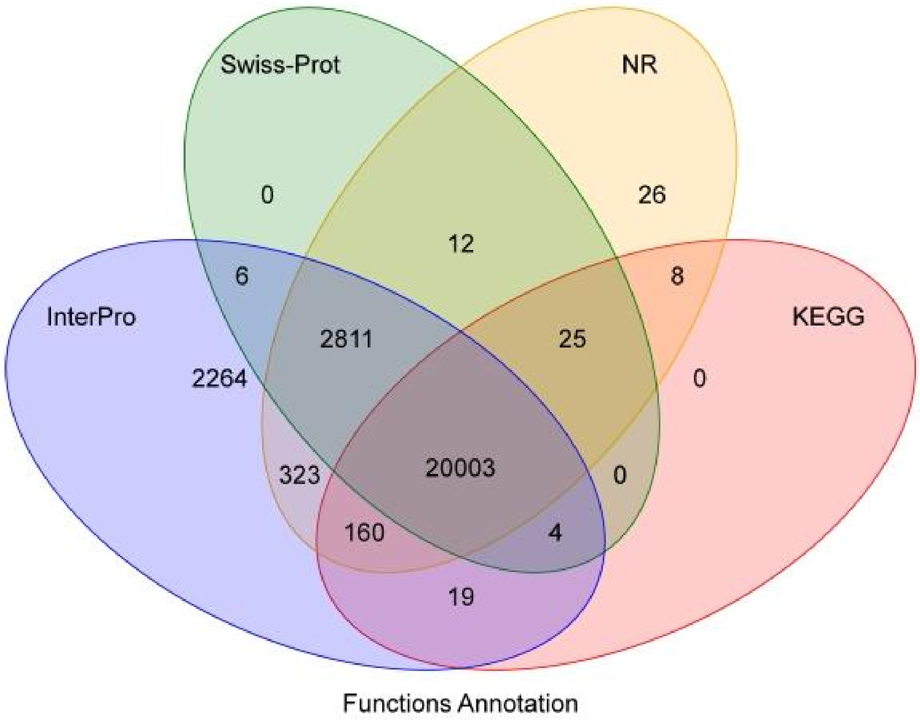

Supplementary Fig. 5 Results of gene functional annotation analysis

Supplementary Table 13 Genes used for gene family clustering in each species.

Species	Abbreviated form	Acronym	Gene
Oryctolagus cuniculus	*O.cuniculus*	Ocu	18,670
Ochotona princeps	*O.princeps*	Opr	18,366
Mus musculus	*M.musculus*	Mmu	23,217
Mus caroli	*M.caroli*	Mca	20,608
Rattus norvegicus	*R.norvegicus*	Rno	22,261
Meriones unguiculatus	*M.unguiculatus*	Mun	20,915
Meriones meridianus	*M.meridianus*	Mme	22,533
Neotoma lepida	*N.lepida*	Nle	20,557
Peromyscus maniculatus	*P.maniculatus*	Pma	21,221
Microtus ochrogaster	*M.ochrogaster*	Moc	19,894
Cricetulus griseus	*C.griseus*	Cgr	21,791
Mesocricetus auratus	*M.auratus*	Mau	18,724
Nannospalax galili	*N.galili*	Nga	20,199
Orientallactaga sibirica	*O.sibirica*	Osi	21,246
Jaculus jaculus	*J.jaculus*	Jja	18,653
Dipus sagitta	*D.sagitta*	Dsa	23,482
Dipodomys ordii	*D.ordii*	Dor	19,262
Castor canadensis	*C.canadensis*	Cca	20,340
Marmota marmota	*M.marmota*	Mma	20,232
Ictidomys tridecemlineatus	*I.tridecemlineatus*	Itr	19,720
Urocitellus parryii	*U.parryii*	Upa	19,133
Chinchilla lanigera	*C.lanigera*	Cla	18,367
Octodon degus	*O.degus*	Ode	20,409
Cavia porcellus	*C.porcellus*	Cpo	19,614
Heterocephalus glaber	*H.glaber*	Hgl	19,400
Fukomys damarensis	*F.damarensis*	Fda	19,283
Monodelphis domestica	*M.domestica*	Mdo	19,505

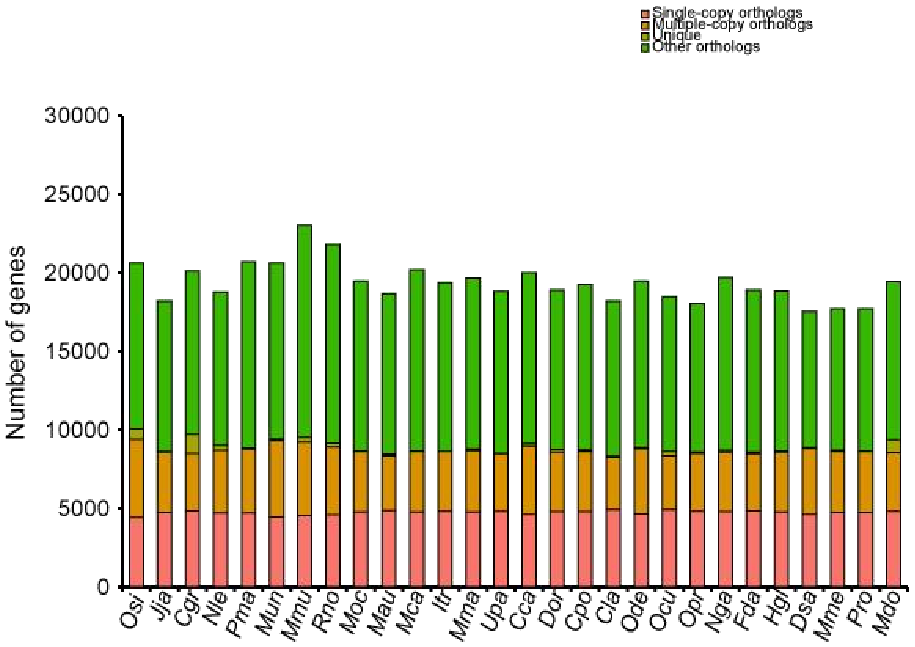

Supplementary Fig. 6 Distribution of genes among species

Note: The horizontal axis represents the 28 species, and the vertical axis represents the number of corresponding genes. Pink represents the number of single-copy gene families, orange represents the number of multiple-copy gene families, cyan represents the number of gene families unique to the corresponding species, and green represents the number of gene families other than those in the above classification.

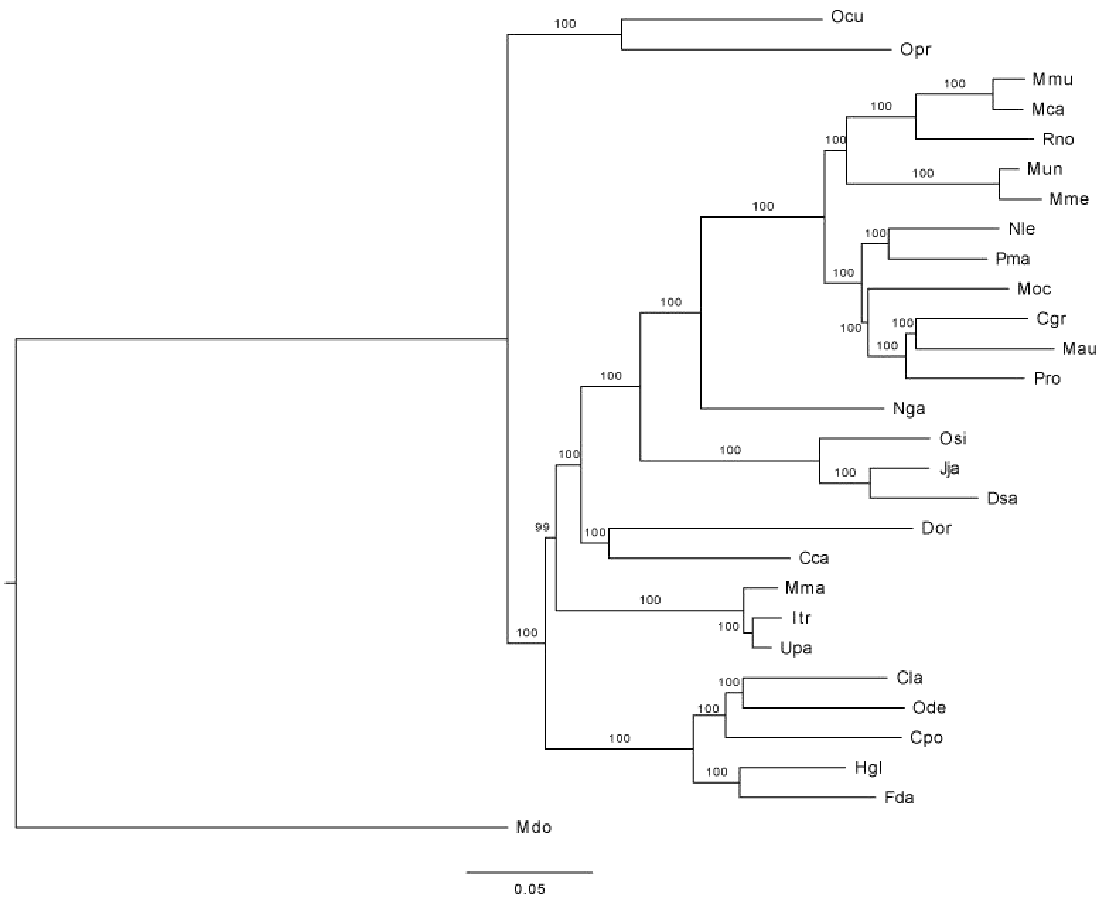

Supplementary Fig. 7 Phylogenetic tree of 28 species.

Supplementary Table 14 The Gene Ontology (GO) and Kyoto Encyclopedia of Genes and Genomes (KEGG) enrichment analysis of gained (green) and lost (red) gene families.

Please refer to “Supplementary Table 14” in the supplementary Excel file for details

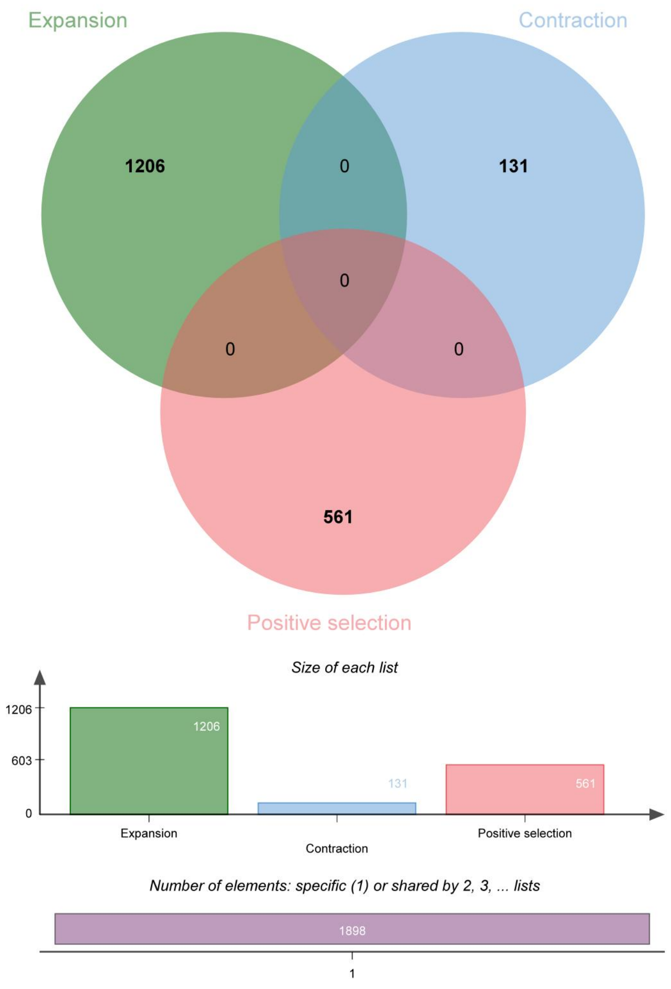

Supplementary Fig. 8 Venn diagram for the three datasets (expansion, contraction, and positive selection)

Supplementary Table 15 Enriched GO terms related to arid adaptation

GO ID	Description	GO Class	P value
GO:0000077	DNA damage checkpoint	BP	0.0066539
GO:0003407	neural retina development	BP	0.0029965
GO:0004497	monooxygenase activity	MF	0.018857362
GO:0004908	interleukin-1 receptor activity	MF	0.000146928
GO:0004954	prostanoid receptor activity	MF	0.038445
GO:0004955	prostaglandin receptor activity	MF	0.026169
GO:0004956	prostaglandin D receptor activity	MF	0.013032
GO:0004970	ionotropic glutamate receptor activity	MF	0.013362
GO:0005230	extracellular ligand-gated ion channel activity	MF	0.010361856
GO:0005231	excitatory extracellular ligand-gated ion channel activity	MF	0.047823
GO:0005234	extracellular-glutamate-gated ion channel activity	MF	0.021308
GO:0005509	calcium ion binding	MF	0.0032832
GO:0005524	ATP binding	MF	0.026250333
GO:0006811	ion transport	BP	0.021087703
GO:0007250	activation of NF-kappaB-inducing kinase activity	BP	0.021935
GO:0008066	glutamate receptor activity	MF	0.013362
GO:0008270	zinc ion binding	MF	0.012268
GO:0008289	lipid binding	MF	0.007510266
GO:0010842	retina layer formation	BP	0.0029965
GO:0016125	sterol metabolic process	BP	0.034523
GO:0016491	oxidoreductase activity	MF	0.012803121
GO:0016705	oxidoreductase activity, acting on paired donors, with incorporation or reduction of molecular oxygen	MF	0.015812843
GO:0019966	interleukin-1 binding	MF	0.031928
GO:0022843	voltage-gated cation channel activity	MF	0.024510225
GO:0022890	inorganic cation transmembrane transporter activity	MF	0.030033567
GO:0030001	metal ion transport	BP	0.016989447
GO:0031098	stress-activated protein kinase signaling cascade	BP	0.021935
GO:0031570	DNA integrity checkpoint	BP	0.0080531

Supplementary Table (continued) 15 Enriched GO terms related to arid adaptation

GO ID	Description	GO Class	P value
GO:0031573	intra-S DNA damage checkpoint	BP	0.038032
GO:0032872	regulation of stress-activated MAPK cascade	BP	0.021935
GO:0034178	toll-like receptor 13 signaling pathway	BP	0.021834
GO:0043167	ion binding	MF	0.045459569
GO:0045454	cell redox homeostasis	BP	0.005037129
GO:0046873	metal ion transmembrane transporter activity	MF	0.009193535
GO:0047750	cholestenol delta-isomerase activity	MF	0.0058153
GO:0051403	stress-activated MAPK cascade	BP	0.021935
GO:0055114	oxidation–reduction process	BP	0.009112373
GO:0071356	cellular response to tumor necrosis factor	BP	0.045907
GO:0071902	positive regulation of protein serine/threonine kinase activity	BP	0.035803
GO:0080134	regulation of response to stress	BP	0.016124

Supplementary Table 16 Enriched KEGG pathways related to arid adaptation

Map ID	Map Title	P value	Adjusted P value
map03460	Fanconi anemia pathway	0.00350937	0.159927024
map00564	Glycerophospholipid metabolism	0.032946034	0.437119549
map00561	Glycerolipid metabolism	0.009904934	0.315967401
map00020	Citrate cycle (TCA cycle)	0.034738247	0.437119549
map00250	Alanine, aspartate and glutamate metabolism	0.004552798	0.181542827
map00350	Tyrosine metabolism	0.017983214	0.409760365
map00052	Galactose metabolism	0.022733441	0.437119549
map01200	Carbon metabolism	0.027803584	0.437119549
map00500	Starch and sucrose metabolism	0.031934919	0.437119549

Supplementary Table 17 Enriched GO terms related to water metabolism in Siberian jerboa

GO_ID	GO_Term	GO_Class	P value
GO:0006664	glycolipid metabolic process	BP	0.018068409
GO:0006683	galactosylceramide catabolic process	BP	0.012439667
GO:0008233	peptidase activity	MF	0.000466922
GO:0008289	lipid binding	MF	0.008625692
GO:0009062	fatty acid catabolic process	BP	0.012439667
GO:0009074	aromatic amino acid family catabolic process	BP	0.020089022
GO:0016042	lipid catabolic process	BP	0.013153473
GO:0016310	phosphorylation	BP	0.010866586
GO:0016485	protein processing	BP	0.007103115
GO:0016598	protein arginylation	BP	0.047017615
GO:0016627	oxidoreductase activity, acting on the CH-CH group of donors	MF	0.000339349
GO:0016705	oxidoreductase activity, acting on paired donors, with incorporation or reduction of molecular oxygen	MF	0.000404953
GO:0016709	oxidoreductase activity, acting on paired donors, with incorporation or reduction of molecular oxygen, NAD(P)H as one donor, and incorporation of one atom of oxygen	MF	0.001331649
GO:0016712	oxidoreductase activity, acting on paired donors, with incorporation or reduction of molecular oxygen, reduced flavin or flavoprotein as one donor, and incorporation of one atom of oxygen	MF	0.046513794
GO:0016787	hydrolase activity	MF	0.007495503
GO:0016936	galactoside binding	MF	0.047017615
GO:0019543	propionate catabolic process	BP	0.047017615
GO:0030258	lipid modification	BP	0.022108665
GO:0030674	protein binding, bridging	MF	0.02081721
GO:0044242	cellular lipid catabolic process	BP	0.002472409
GO:0044255	cellular lipid metabolic process	BP	0.02445777
GO:0006664	glycolipid metabolic process	BP	0.018068409
GO:0044710	single-organism metabolic process	BP	0.009536037
GO:0045444	fat cell differentiation	BP	0.047017615
GO:0050660	flavin adenine dinucleotide binding	MF	0.018326812
GO:0055114	oxidation–reduction process	BP	0.016172823
GO:2001070	starch binding	MF	0.047017615

Supplementary Table (continued) 17 Enriched GO terms related to water metabolism in Siberian jerboa

GO_ID	GO_Term	GO_Class	P value
GO:0003824	catalytic activity	MF	3.45E-05
GO:0004057	arginyltransferase activity	MF	0.047017615
GO:0004175	endopeptidase activity	MF	0.000339033
GO:0004714	transmembrane receptor protein tyrosine kinase activity	MF	0.000985642
GO:0004974	leukotriene receptor activity	MF	0.012164966
GO:0005515	protein binding	MF	4.81E-07
GO:0006468	protein phosphorylation	BP	0.012749539
GO:0006508	proteolysis	BP	0.000648955
GO:0006559	L-phenylalanine catabolic process	BP	0.047017615
GO:0006570	tyrosine metabolic process	BP	0.047017615
GO:0006629	lipid metabolic process	BP	0.019495718
GO:0006644	phospholipid metabolic process	BP	0.025768537
GO:0006650	glycerophospholipid metabolic process	BP	0.028025027

Supplementary Table 18 Enriched KEGG pathways related to water metabolism in Siberian jerboa

Map ID	Map Title	P value	Adjusted P value
map00630	Glyoxylate and dicarboxylate metabolism	0.030420048	0.541137374
map00643	Styrene degradation	0.048816507	0.596500553
map03320	PPAR signaling pathway	0.043574888	0.596500553
map04920	Adipocytokine signaling pathway	0.047047659	0.596500553
map04931	Insulin resistance	0.00493477	0.24920587
map04977	Vitamin digestion and absorption	0.010653464	0.319791631

Supplementary Table 19 Enriched GO terms related to limb enlargement in Siberian jerboa

GO_ID	GO_Term	GO_Class	P value
GO:0003774	motor activity	MF	0.045499004
GO:0005856	cytoskeleton	CC	0.016230626
GO:0005884	actin filament	CC	0.031597638
GO:0009790	embryo development	BP	0.002472409
GO:0015629	actin cytoskeleton	CC	0.002746749
GO:0016459	myosin complex	CC	0.00326019
GO:0022603	regulation of anatomical structure morphogenesis	BP	0.027966814
GO:0043589	skin morphogenesis	BP	0.047017615
GO:0051017	actin filament bundle assembly	BP	0.001863633
GO:0051764	actin crosslink formation	BP	0.001863633
GO:0071203	WASH complex	CC	0.008771309

Supplementary Table 20 Enriched GO terms related to adaptation to arid environments

GO_ID	GO_Term	GO_Class	P value
GO:0000976	transcription regulatory region sequence-specific DNA binding	MF	8.19E-09
GO:0004962	endothelin receptor activity	MF	0.051592
GO:0004977	melanocortin receptor activity	MF	0.026477
GO:0004980	melanocyte-stimulating hormone receptor activity	MF	0.014397
GO:0005000	vasopressin receptor activity	MF	0.16886
GO:0005976	polysaccharide metabolic process	BP	0.035813
GO:0005978	glycogen biosynthetic process	BP	0.0074174
GO:0006355	regulation of transcription, DNA-dependent	BP	2.00E-06
GO:0006412	translation	BP	1.08E-52
GO:0006511	ubiquitin-dependent protein catabolic process	BP	1.32E-54
GO:0007601	visual perception	BP	0.6255
GO:0016787	hydrolase activity	MF	3.93E-05
GO:0031326	regulation of cellular biosynthetic process	BP	9.92E-06
GO:0046872	metal ion binding	MF	0.00034834
GO:0050794	regulation of cellular process	BP	0.00060767

Supplementary Table 21 Enriched KEGG pathways related to adaptation to arid environments

Map ID	Map Title	P value	Adjusted P value
map05322	Systemic lupus erythematosus	2.17E-161	3.48E-159
map03010	Ribosome	7.64E-141	6.11E-139
map05330	Allograft rejection	5.55E-120	2.96E-118
map05320	Autoimmune thyroid disease	5.06E-113	2.02E-111
map05150	*Staphylococcus aureus* infection	6.83E-71	2.19E-69
map05416	Viral myocarditis	2.83E-68	7.56E-67
map04940	Type I diabetes mellitus	1.34E-67	3.07E-66
map04672	Intestinal immune network for IgA production	1.27E-47	2.53E-46
map04612	Antigen processing and presentation	4.69E-46	8.33E-45
map04740	Olfactory transduction	7.83E-41	1.25E-39
map05034	Alcoholism	1.53E-39	2.22E-38
map05332	Graft-versus-host disease	9.68E-32	1.29E-30
map05310	Asthma	2.18E-30	2.68E-29
map05202	Transcriptional misregulation in cancer	5.59E-30	6.38E-29
map05323	Rheumatoid arthritis	1.13E-26	1.21E-25
map04650	Natural killer cell mediated cytotoxicity	1.01E-23	1.01E-22
map05162	Measles	1.28E-22	1.20E-21
map04064	NF-kappa B signaling pathway	9.31E-22	8.28E-21
map05203	Viral carcinogenesis	8.18E-21	6.89E-20
map05169	Epstein–Barr virus infection	3.13E-19	2.51E-18
map04145	Phagosome	5.25E-19	4.00E-18
map04514	Cell adhesion molecules	1.46E-18	1.06E-17
map04020	Calcium signaling pathway	4.38E-12	3.05E-11
map00072	Synthesis and degradation of ketone bodies	9.13E-12	6.09E-11
map05414	Dilated cardiomyopathy	3.19E-10	2.04E-09
map04072	Phospholipase D signaling pathway	3.61E-10	2.22E-09
map05321	Inflammatory bowel disease	5.70E-10	3.38E-09
map05143	African trypanosomiasis	1.31E-09	7.47E-09

Supplementary Table (continued) 21 Enriched KEGG pathways related to adaptation to arid environments

Map ID	Map Title	P value	Adjusted P value
map05340	Primary immunodeficiency	5.58E-09	3.08E-08
map00650	Butanoate metabolism	3.61E-08	1.93E-07
map05144	Malaria	5.79E-08	2.99E-07
map05140	Leishmaniasis	7.99E-07	3.93E-06
map04015	Rap1 signaling pathway	8.11E-07	3.93E-06
map05166	HTLV-I infection	2.64E-05	0.000124047
map04640	Hematopoietic cell lineage	2.99E-05	0.000136658
map04212	Longevity regulating pathway - worm	3.54E-05	0.000157156
map04664	Fc epsilon RI signaling pathway	0.000141385	0.000595304
map04550	Signaling pathways regulating pluripotency of stem cells	0.000651453	0.002605811
map05142	Chagas disease (American trypanosomiasis)	0.000823738	0.003214587
map04666	Fc gamma R-mediated phagocytosis	0.001247553	0.004435746
map04662	B-cell receptor signaling pathway	0.001599469	0.005445
map04014	Ras signaling pathway	0.002054359	0.006708112
map04978	Mineral absorption	0.00245845	0.007712784
map05146	Amoebiasis	0.003845134	0.01139299
map00591	Linoleic acid metabolism	0.004983961	0.014239887
map05210	Colorectal cancer	0.005401599	0.015162384
map04660	T-cell receptor signaling pathway	0.007346164	0.019896701
map05212	Pancreatic cancer	0.007461263	0.019896701
map05134	Legionellosis	0.028590366	0.064428994
map04151	PI3K-Akt signaling pathway	0.036170098	0.076147574
map04380	Osteoclast differentiation	0.037078824	0.077046907
map04913	Ovarian steroidogenesis	0.043731951	0.08857104
map05152	Tuberculosis	0.046574513	0.091999038
map00140	Steroid hormone biosynthesis	0.049455975	0.094337449

Supplementary Table 22 Raw transcriptome data from wild rodents

Sample	Raw reads	Clean reads	Clean base （Gb）	Error rate（%）	Q20 （%）	Q30（%）	GC content （%）
Asi01K	51905588	51559958	7.73	0.03	97.96	94.17	48.66
Asi02K	50761074	48406920	7.26	0.03	97.82	93.81	48.94
Asi03K	48798428	46477720	6.97	0.03	97.35	92.93	48.71
G601K	54930336	54197010	8.13	0.02	98.05	94.31	49.46
G602K	51061766	50455640	7.57	0.02	98.05	94.32	49.72
G603K	47335096	46644714	7.0	0.02	98.17	94.56	49.85
H401K	44317370	43765050	6.56	0.02	98.12	94.42	49.98
H402K	48885430	48644184	7.3	0.03	97.94	94.19	49.43
H403K	47146434	46581300	6.99	0.02	98.1	94.48	49.61
M2301K	47288706	46753812	7.01	0.02	98.18	94.62	49.73
M2302K	47192284	46933068	7.04	0.02	98.3	94.82	49.22
M2303K	46890342	46684202	7.0	0.03	97.89	94.1	48.97

Note: Sample represents the name of the sample; Raw reads is the number of raw data reads; Clean reads is the number of reads filtered from the original data; Clean base is the base number after filtering the original data; Error rate is the sequencing error rate; Q20 is the percentage of bases with a Phred value greater than 20 in the total number of bases; Q30 is the percentage of bases with a Phred value greater than 30 in the total number of bases; GC Content is the percentage of G and C among the four bases in the clean reads.

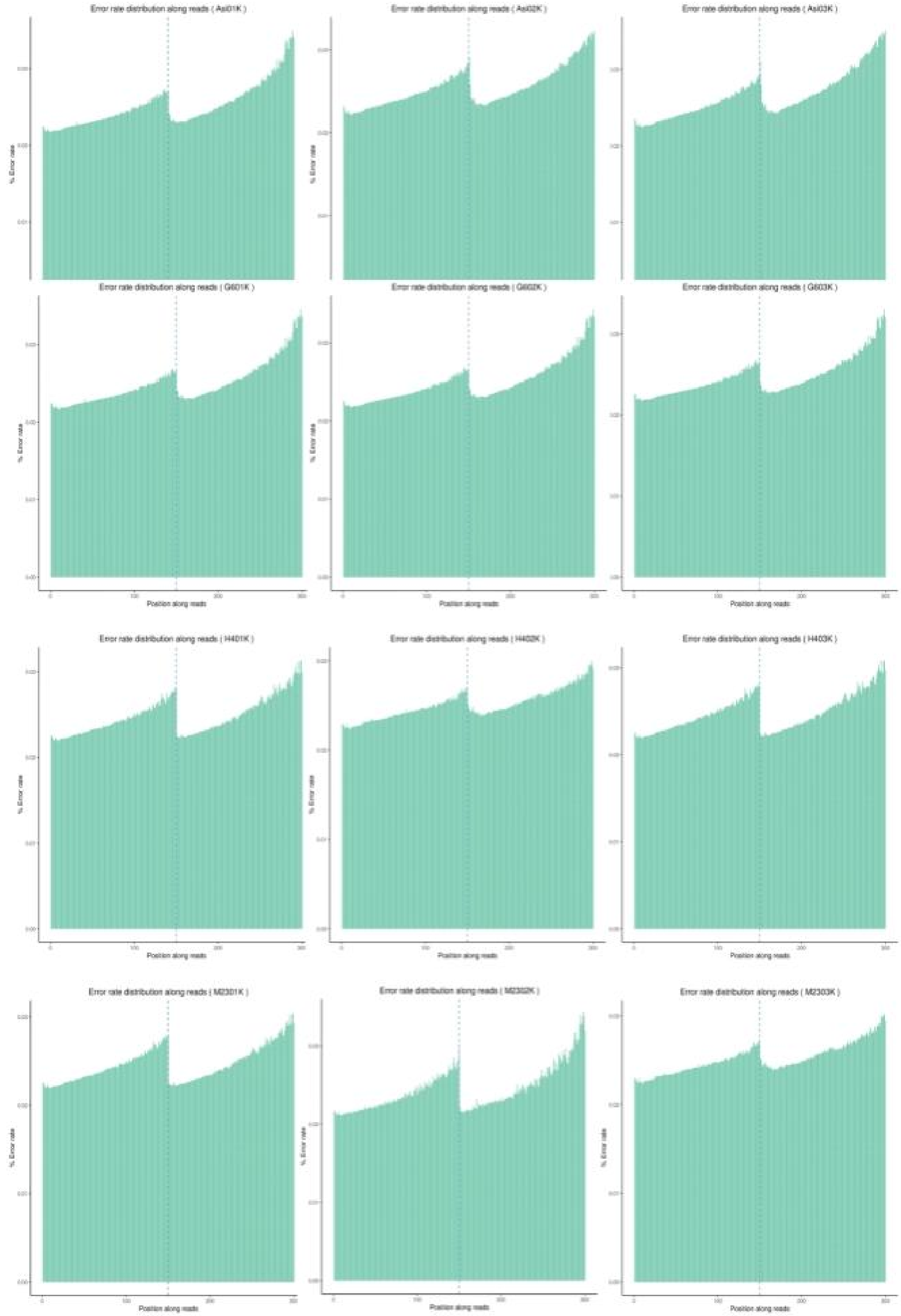

Supplementary Fig. 9 Error rate distributions of the sequencing data

Note: The horizontal axis represents the base position of the reads, whereas the vertical axis represents the single-base error rate.

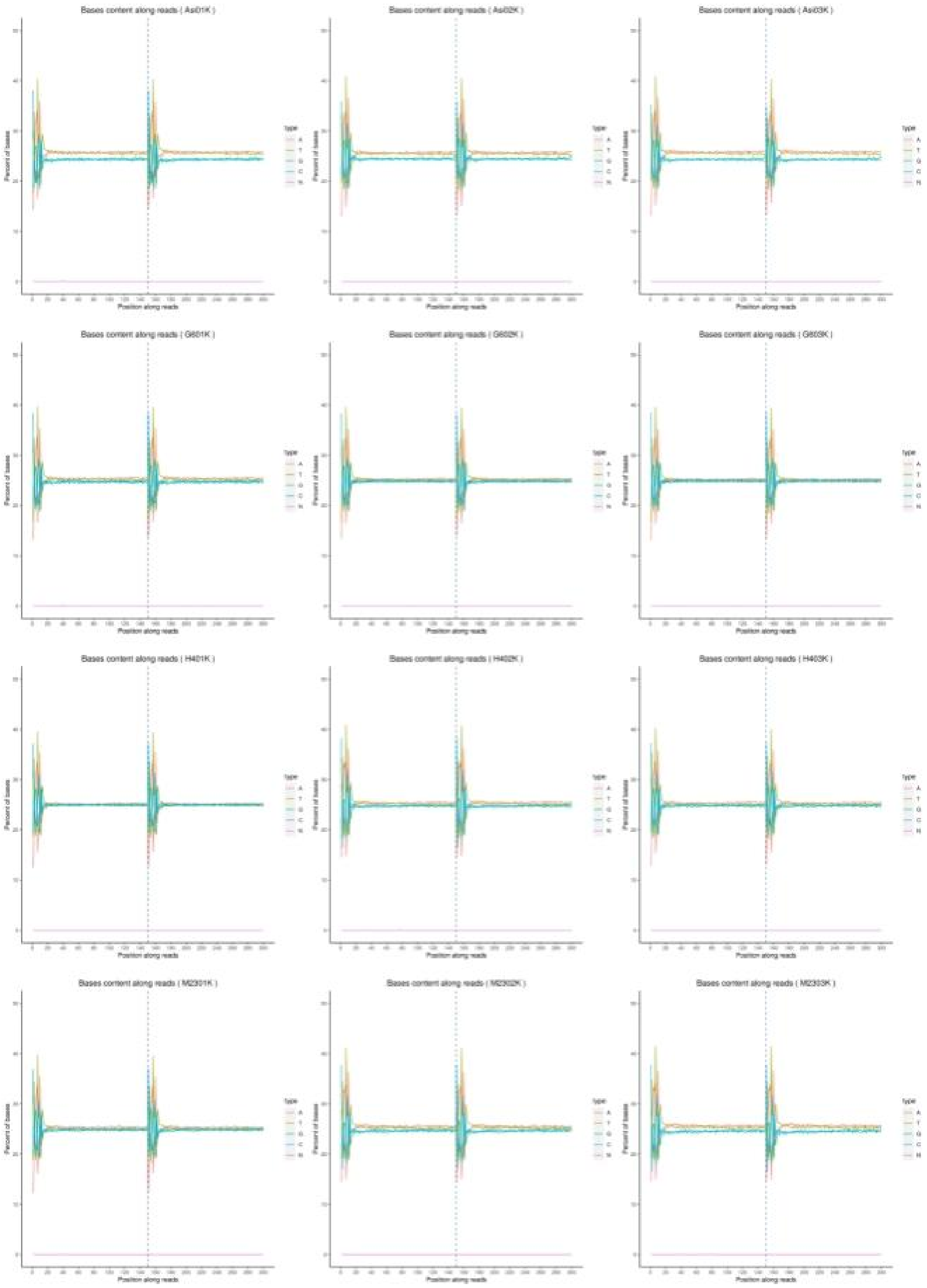

Supplementary Fig. 10 The distribution of GC content in the kidney samples of Siberian jerboa

Note: The horizontal axis represents the base position of the reads, whereas the vertical axis represents the percentage of the five base types in ATGCN. Red represents A, light green represents T, green represents G, blue represents C, and purple represents N.

Supplementary Table 23 Reads compared with the reference genome

Sample	Total Reads	Total Map	Unique Map	Multi Map
Asi01K	51559958	49301549(95.62%)	38817872(75.29%)	10483677(20.33%)
Asi02K	48406920	46012950(95.05%)	35721708(73.79%)	10291242(21.26%)
Asi03K	46477720	43617576(93.85%)	34375654(73.96%)	9241922(19.88%)
G601K	54197010	51521931(95.06%)	42070708(77.63%)	9451223(17.44%)
G602K	50455640	48059155(95.25%)	39857304(78.99%)	8201851(16.26%)
G603K	46644714	44484951(95.37%)	37192853(79.74%)	7292098(15.63%)
H401K	43765050	40527900(92.6%)	34314909(78.41%)	6212991(14.2%)
H402K	48644184	44900091(92.3%)	38438610(79.02%)	6461481(13.28%)
H403K	46581300	42956095(92.22%)	35605153(76.44%)	7350942(15.78%)
M2301K	46753812	42878739(91.71%)	34911836(74.67%)	7966903(17.04%)
M2302K	46933068	42676883(90.93%)	34417818(73.33%)	8259065(17.6%)
M2303K	46684202	42533657(91.11%)	35055437(75.09%)	7478220(16.02%)

Note: Total reads: Statistics of the number of sequencing sequences filtered by sequencing data (Clean data). Total mapped: Statistics of the number of sequencing sequences that can be located on the genome. Multiple mapped: the number of sequencing sequences with multiple alignment positions on the reference sequence. Unique mapped: counts the number of sequencing sequences with unique alignment positions on the reference sequence.

Supplementary Table 24 Results of the GO enrichment analysis of the top DEGs in the kidney of Siberian jerboa in the wild

GO_ID	GO_Term	GO_Class	P value
GO:0048037	cofactor binding	MF	0.00000636
GO:0061134	peptidase regulator activity	MF	0.000105237
GO:0061135	endopeptidase regulator activity	MF	0.0000249
GO:0070279	vitamin B6 binding	MF	0.001942792
GO:0098609	cell–cell adhesion	BP	0.000158235
GO:0098742	cell–cell adhesion via plasma-membrane adhesion molecules	BP	0.000137594

Supplementary Table (continued) 24 Results of the GO Enrichment Analysis of the DEGs in the kidney of Siberian jerboa

GO_ID	GO_Term	GO_Class	P value
GO:0004222	metalloendopeptidase activity	MF	0.000329741
GO:0004857	enzyme inhibitor activity	MF	0.000537894
GO:0004866	endopeptidase inhibitor activity	MF	0.0000249
GO:0005201	Extracellular matrix structural constituent	MF	0.00000089
GO:0005342	organic acid transmembrane transporter activity	MF	0.001325881
GO:0005506	iron ion binding	MF	0.0000229
GO:0005509	calcium ion binding	MF	1.94E-11
GO:0005576	extracellular region	CC	7.11E-08
GO:0005581	collagen trimer	CC	0.000000407
GO:0007155	cell adhesion	BP	0.000000447
GO:0007156	homophilic cell adhesion via plasma membrane adhesion molecules	BP	0.000137594
GO:0008237	metallopeptidase activity	MF	0.001032527
GO:0008514	organic anion transmembrane transporter activity	MF	0.001729022
GO:0015002	heme-copper terminal oxidase activity	MF	0.002218725
GO:0015081	sodium ion transmembrane transporter activity	MF	0.001479108
GO:0015293	symporter activity	MF	0.000258705
GO:0015294	solute:cation symporter activity	MF	0.000258705
GO:0015370	solute:sodium symporter activity	MF	0.0000963
GO:0016705	oxidoreductase activity, acting on paired donors, with incorporation or reduction of molecular oxygen	MF	0.000315412
GO:0019842	vitamin binding	MF	0.001904923
GO:0020037	heme binding	MF	0.00003
GO:0022610	biological adhesion	BP	0.000000447
GO:0030170	pyridoxal phosphate binding	MF	0.001942792
GO:0030414	peptidase inhibitor activity	MF	0.000105237
GO:0031012	extracellular matrix	CC	0.00000783
GO:0044421	extracellular region part	CC	0.000000159
GO:0046906	tetrapyrrole binding	MF	0.0000444
GO:0046943	carboxylic acid transmembrane transporter activity	MF	0.001325881

Supplementary Table 25 Results of the KEGG Enrichment analysis of the DEGs in the kidney of Siberian jerboa

Map ID	Map title	P value	Adiusted P value
rno04512	ECM-receptor interaction	2.10E-14	6.16E-12
rno04974	Protein digestion and absorption	2.30E-13	3.37E-11
rno04510	Focal adhesion	3.95E-10	3.86E-08
rno04151	PI3K-Akt signaling pathway	8.86E-07	6.49E-05
rno03320	PPAR signaling pathway	1.21E-05	7.11E-04
rno00980	Metabolism of xenobiotics by cytochrome P450	8.46E-05	4.13E-03
rno04923	Regulation of lipolysis in adipocytes	1.15E-04	4.80E-03
rno04913	Ovarian steroidogenesis	4.80E-04	1.76E-02
rno00140	Steroid hormone biosynthesis	7.27E-04	2.25E-02
rno05165	Human papillomavirus infection	7.68E-04	2.25E-02
rno00260	Glycine, serine and threonine metabolism	9.11E-04	2.26E-02
rno05204	Chemical carcinogenesis	9.28E-04	2.26E-02
rno04270	Vascular smooth muscle contraction	1.13E-03	2.55E-02
rno04022	cGMP-PKG signaling pathway	1.48E-03	3.10E-02
rno04260	Cardiac muscle contraction	1.80E-03	3.52E-02

Supplementary Table 26 Blood biochemical indicators of Siberian jerboa under WS conditions

Index	Unit	Average Value		P value
		WSAsi	Asi	
Albumin	g/L	24.5±4.226	28.25±4.986	0.018*
Total protein	g/L	35.15±6.031	36.15±6.274	0.49
Globulin	g/L	10.65±2.145	7.9±1.722	0.082
Globulin ratio	g/L	2.03±0.418	3.33±0.750	0.019*
Glucose	mmol/L	11.653±2.533	11.616±0.923	0.63
Urea nitrogen	mmol/L	11.173±0.803	20.64±1.755	0**
Inorganic phosphorus	mmol/L	1.668±1.013	0.261±0.052	0.135
Cholesterol	mmol/L	8.121±1.224	9.555±0.5	0.25
Total bilirubin	umol/L	5.037±0.92	4.189±0.626	0.42
Creatinine	umol/L	54.778±6.839	47.6±4.037	0.289
Urea nitrogen/creatinine ratio		55.6±7.893	117.8±15.932	0.002**
Alkaline phosphatase	U/L	22.444±1.872	22.3±2.135	0.971
Alanine aminotransferase	U/L	49.181±10.527	228.5±66.395	0.041*
Amylase	U/L	790.111±91.051	964.5±110.212	0.454
Creatine kinase	U/L	1729.444±215.228	2614±506.571	0.259
Nitrogen		10	10	

Note: In the table, * represents significant differences ($p<0.05$), and ** represents extremely significant differences ($p<0.01$). WS, water deprivation stress.

Supplementary Table 27 Aridity stress experiment transcriptome analysis results

Sample	Raw reads	Clean reads	Clean base （G）	Error rate（%）	Q20 （%）	Q30（%）	GC content （%）
Asi_1K	104281744	101301418	15.2	0.02	98.61	95.37	47.79
Asi_2K	83334882	80547096	12.08	0.02	98.43	94.93	47.81
Asi_3K	83064222	81930982	12.29	0.03	96.99	91.95	47.5
Asi_4K	104281960	100804936	15.12	0.02	98.54	95.26	48.37
WSAsi_1K	81264336	77723184	11.66	0.03	98.09	94.06	45.91
WSAsi_2K	82297086	9278484	11.39	0.03	97	91.3	47.44
WSAsi_3K	97566324	94041236	14.11	0.02	98.33	94.63	47.76
WSAsi_4K	83248156	82290754	12.34	0.03	97.7	93.58	47.54

Note: Sample represents the name of the sample; Raw reads is the number of raw data reads; Clean reads is the number of reads filtered from the original data; Clean base is the base number after filtering the original data; Error rate is the sequencing error rate; Q20 is the percentage of bases with a Phred value greater than 20 in the total number of bases; Q30 is the percentage of bases with a Phred value greater than 30 in the total number of bases; GC content is the percentage of G and C among the four bases in the clean reads.

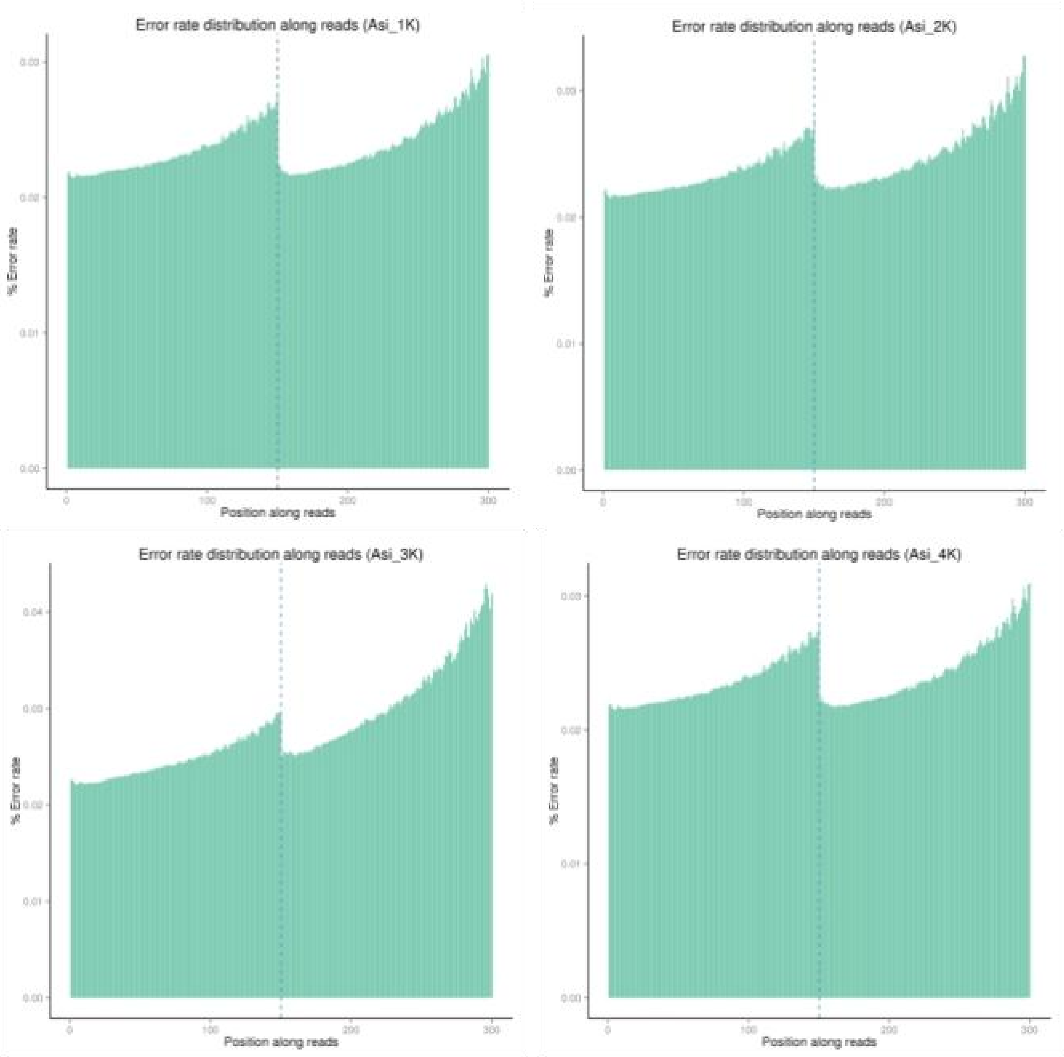

Supplementary Fig. 11 CK error rate distribution of the sequencing data

Note: The horizontal axis represents the base position of the reads, whereas the vertical axis represents the single-base error rate.

Note: CK, control group.

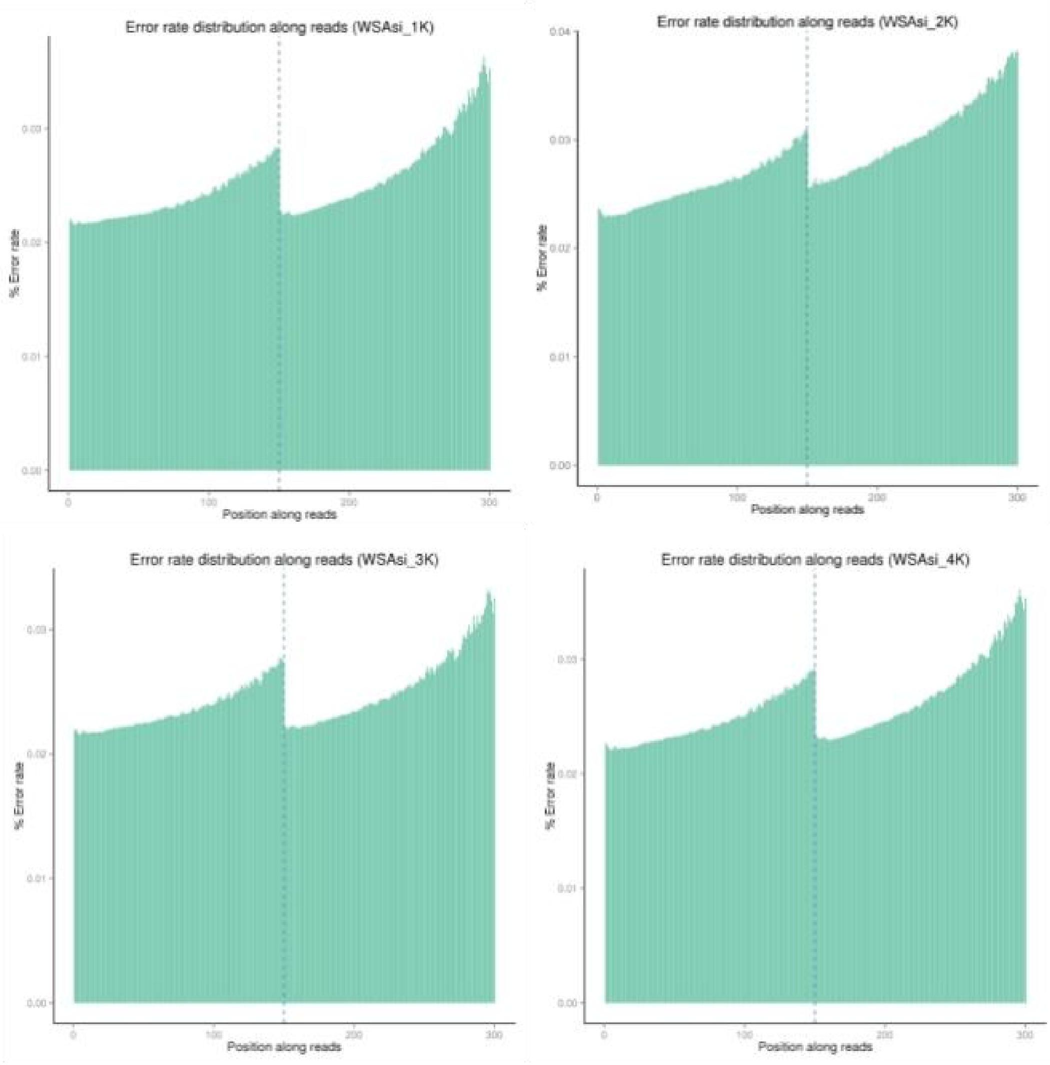

Supplementary Fig. 12 WS error rate distribution of sequencing data

Note: The horizontal axis in the figure represents the base position of the reads, whereas the vertical axis represents the single-base error rate.

Note: WS, water deprivation stress.

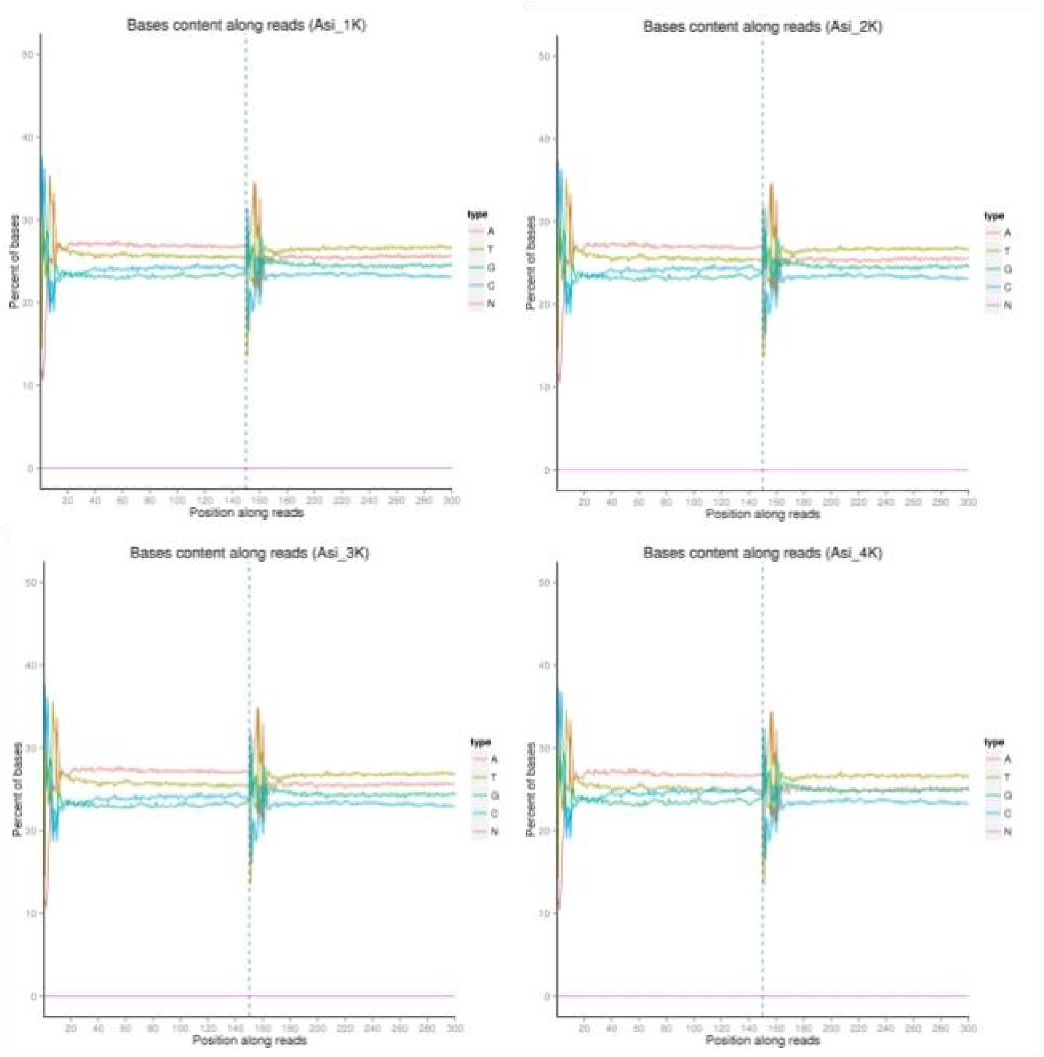

Supplementary Fig. 13 GC content distribution of CK kidney samples

Note: The horizontal axis represents the base position of the reads, whereas the vertical axis represents the percentage of the five base types in ATGCN. Red represents A, light green represents T, green represents G, blue represents C, and purple represents N.

Note: CK, control group.

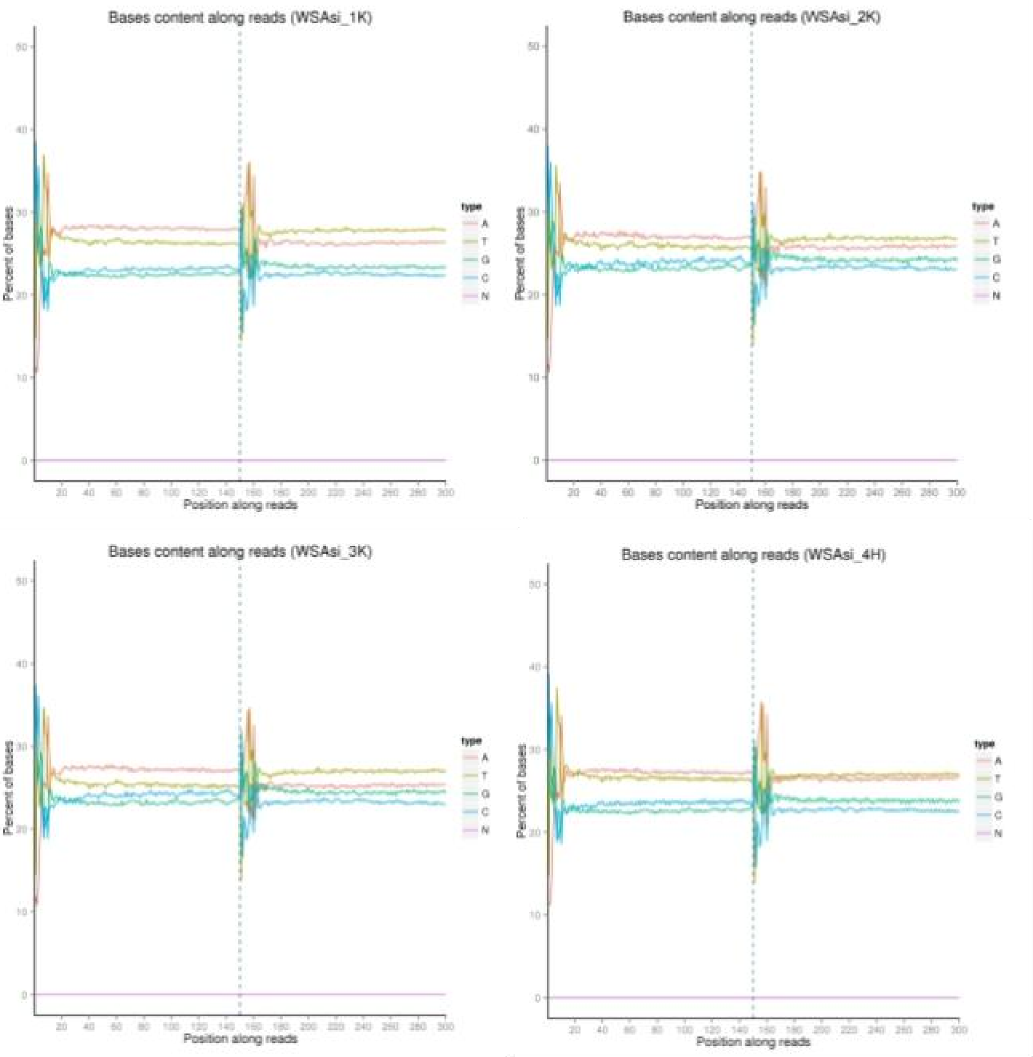

Supplementary Fig. 14 GC content distribution of WS kidney samples

Note: The horizontal axis represents the base position of reads, while the vertical axis represents the percentage of five base types in ATGCN. Red represents A, light green represents T, green represents G, blue represents C, and purple represents N.

Note: WS, water deprivation stress.

Supplementary Table 28 Results of KEGG enrichment analysis of DEGs in the kidney of Siberian jerboa (CK vs. WS)

Map ID	Map title	P value	Adjusted P value
rno04723	Retrograde endocannabinoid signaling	0.00003	0.00471
rno04261	Adrenergic signaling in cardiomyocytes	0.00004	0.00471
rno04720	Long-term potentiation	0.00012	0.00924
rno05414	Dilated cardiomyopathy	0.00018	0.01030
rno04727	GABAergic synapse	0.00022	0.01030
rno05410	Hypertrophic cardiomyopathy (HCM)	0.00035	0.01376
rno04024	cAMP signaling pathway	0.00058	0.01574
rno04921	Oxytocin signaling pathway	0.00059	0.01574
rno00072	Synthesis and degradation of ketone bodies	0.00060	0.01574
rno00330	Arginine and proline metabolism	0.00073	0.01731
rno00650	Butanoate metabolism	0.00123	0.02308
rno04911	Insulin secretion	0.00126	0.02308
rno04020	Calcium signaling pathway	0.00127	0.02308
rno04970	Salivary secretion	0.00137	0.02309
rno00982	Drug metabolism - cytochrome P450	0.00200	0.02827
rno04918	Thyroid hormone synthesis	0.00200	0.02827
rno04724	Glutamatergic synapse	0.00209	0.02827
rno05032	Morphine addiction	0.00216	0.02827
rno05033	Nicotine addiction	0.00236	0.02934
rno05412	Arrhythmogenic right ventricular cardiomyopathy (ARVC)	0.00325	0.03664
rno04972	Pancreatic secretion	0.00326	0.03664
rno04512	ECM-receptor interaction	0.00344	0.03693
rno04725	Cholinergic synapse	0.00380	0.03899
rno00480	Glutathione metabolism	0.00475	0.04667
rno04611	Platelet activation	0.00711	0.06601
rno04713	Circadian entrainment	0.00752	0.06601
rno04260	Cardiac muscle contraction	0.00759	0.06601
rno04114	Oocyte meiosis	0.00783	0.06601

Note: CK, control group; WS, water deprivation stress.

Supplementary Table. (continued) 28 Results of KEGG enrichment analysis of DEGs in the kidney of Siberian jerboa (CK vs. WS)

Map ID	Map title	P value	Adjusted P value
rno04614	Renin-angiotensin system	0.00982	0.07991
rno04540	Gap junction	0.01025	0.08061
rno00740	Riboflavin metabolism	0.01069	0.08142
rno05031	Amphetamine addiction	0.01104	0.08144
rno04912	GnRH signaling pathway	0.01182	0.08452
rno04728	Dopaminergic synapse	0.01360	0.09442
rno04270	Vascular smooth muscle contraction	0.01834	0.12367
rno00350	Tyrosine metabolism	0.01899	0.12450
rno04974	Protein digestion and absorption	0.02727	0.17396
rno04726	Serotonergic synapse	0.03915	0.24312
rno04750	Inflammatory mediator regulation of TRP channels	0.04543	0.25012
rno04971	Gastric acid secretion	0.04544	0.25012
rno04610	Complement and coagulation cascades	0.04544	0.25012
rno04976	Bile secretion	0.04544	0.25012
rno04022	cGMP-PKG signaling pathway	0.04557	0.25012

Note: CK, control group; WS, water deprivation stress.

Supplementary Table 29 GO Enrichment analysis results of the positively selected DEGs in Siberian jerboa (CK vs. WS)

GO_ID	GO_Term	GO_Class	P value	Adjusted P value
GO:0055114	oxidation–reduction process	BP	0.00003	0.05889
GO:0006544	glycine metabolic process	BP	0.00888	1.00000
GO:0006304	DNA modification	BP	0.00918	1.00000
GO:0006629	lipid metabolic process	BP	0.00963	1.00000
GO:0031640	killing of cells of other organism	BP	0.03049	1.00000
GO:0044364	disruption of cells of other organism	BP	0.03049	1.00000
GO:0051818	disruption of cells of other organism involved in symbiotic interaction	BP	0.03049	1.00000
GO:0051883	killing of cells in other organism involved in symbiotic interaction	BP	0.03049	1
GO:0006171	cAMP biosynthetic process	BP	0.0322	1
GO:0046058	cAMP metabolic process	BP	0.0322	1
GO:0012502	induction of programmed cell death	BP	0.03642	1
GO:0019051	induction by virus of host apoptotic process	BP	0.03642	1
GO:0052151	positive regulation by symbiont of host apoptotic process	BP	0.03642	1
GO:0052330	positive regulation by organism of programmed cell death in other organism involved in symbiotic interaction	BP	0.03642	1
GO:0052501	positive regulation by organism of apoptotic process in other organism involved in symbiotic interaction	BP	0.03642	1
GO:0060139	positive regulation of apoptotic process by virus	BP	0.03642	1
GO:0044255	cellular lipid metabolic process	BP	0.03687	1
GO:0009187	cyclic nucleotide metabolic process	BP	0.03839	1
GO:0031343	positive regulation of cell killing	BP	0.03642	1
GO:0016491	oxidoreductase activity	MF	0	0.00074
GO:0016705	oxidoreductase activity, acting on paired donors, with incorporation or reduction of molecular oxygen	MF	0.0001	0.15389

Note: CK, control group; WS, water deprivation stress.

Supplementary Table (continued-1)29 GO Enrichment analysis results of the positively selected DEGs in Siberian jerboa (CK vs WS)

GO_ID	GO_Term	Category	P value	Adjusted P value
GO:0005230	extracellular ligand-gated ion channel activity	MF	0.00017	0.19401
GO:0005216	ion channel activity	MF	0.0007	0.31168
GO:0022838	substrate-specific channel activity	MF	0.0007	0.31168
GO:0008241	peptidyl-dipeptidase activity	MF	0.00073	0.31168
GO:0015267	channel activity	MF	0.00073	0.31168
GO:0022803	passive transmembrane transporter activity	MF	0.00073	0.31168
GO:0016651	oxidoreductase activity， acting on NAD(P)H	MF	0.00199	0.47958
GO:0016668	oxidoreductase activity， acting on a sulfur group of donors， NAD(P) as acceptor	MF	0.00205	0.47958
GO:0047134	protein-disulfide reductase activity	MF	0.00205	0.47958
GO:0004675	transmembrane receptor protein serine/threonine kinase activity	MF	0.00546	1
GO:0050662	coenzyme binding	MF	0.00622	1
GO:0048037	cofactor binding	MF	0.00682	1
GO:0016667	oxidoreductase activity， acting on a sulfur group of donors	MF	0.00683	1
GO:0019825	oxygen binding	MF	0.00702	1
GO:0046789	host cell surface receptor binding	MF	0.01059	1
GO:0004560	alpha-L-fucosidase activity	MF	0.02844	1
GO:0015928	fucosidase activity	MF	0.02844	1
GO:0004497	monooxygenase activity	MF	0.03088	1
GO:0003824	catalytic activity	MF	0.03113	1
GO:0022891	substrate-specific transmembrane transporter activity	MF	0.03672	1
GO:0015238	drug transmembrane transporter activity	MF	0.03715	1
GO:0090484	drug transporter activity	MF	0.03715	1
GO:0051287	NAD binding	MF	0.04034	1
GO:0015075	ion transmembrane transporter activity	MF	0.04274	1
GO:0004529	exodeoxyribonuclease activity	MF	0.04379	1
GO:0005509	calcium ion binding	MF	0.04545	1
GO:0043902	positive regulation of multiorganism process	BP	0.03642	1

Note: CK, control group; WS, water deprivation stress.

Supplementary Table 30 GO enrichment analysis results of the postively selected DEGs associated with reproduction in Siberian jerboa (CK vs WS)

GO_ID	GO_Term	GO_Class	P value	Adjusted P value
GO:0044710	single-organism metabolic process	BP	0.00029	0.25472
GO:0044699	single-organism process	BP	0.00033	0.25472
GO:0007339	binding of sperm to zona pellucida	BP	0.00097	0.32417
GO:0009988	cell–cell recognition	BP	0.00097	0.32417
GO:0035036	sperm-egg recognition	BP	0.00097	0.32417
GO:0008037	cell recognition	BP	0.00155	0.45402
GO:0007338	single fertilization	BP	0.00165	0.45402
GO:0009566	fertilization	BP	0.00165	0.45402

Note: CK, control group; WS, water deprivation stress.

Supplementary Table 31 RT-qPCR primer synthesis information

gene number	Primers	Sequence (5'to3')	TM
Internal reference	*Gapdh*-F	TCAGAGTGAACGGATTTGG	63
	Gapdh -R	GGTGGAATCATACTGGAACAT	
1	*Col12a1*-F	AACTCAGGTATCCGAGGA	63
	Col12a1-R	GCACTATGAAGGGCTCTC	
2	*Col5a3*-F	CAGCCAGCCAATCAGTCT	60
	Col5a3-R	TGAAGGAGTCACCAAGGAG	
3	*Mt-nd1*-F	GCATCCAACTCCAAATACG	63
	Mt-nd1-R	TGAAGAATAGGGCGAAAGG	
4	*Lipe*-F	GCAGTTGGAGTTGAGGATG	63
	Lipe-R	GCACACAGGACACTAAGTTT	
5	*Col6a3*-F	GAAACTTAGTGTCCTGTGTG	63
	Col6a3-R	AATGTCAGCAGAGTCTTGT	
6	*Col9a2*-F	TATTCTCCATCAGCCATC	63
	Col9a2-R	CTCACCCTCAACAGATAC	
7	*Maob*-F	TGACACAGGTGGAACTTAATC	63
	Maob-R	TGACTCTACACAAGGACAAC	
8	*Calhm3*-F	ATGTGGACGGTGGAAGTG	60
	Calhm3-R	AGGTGCTTAGGAGGTGGT	
9	*Cyp4a11*-F	CCTTGATGACCCTGGACAC	60
	Cyp4a11-R	AACAGCCTGGAGATAAGATTGG	
10	*Myoz1*-F	GTGGAACTCGGCATTGAC	63
	Myoz1-R	AACTTAGGCATCTGGAAGGT	

Supplementary Table (continued-1) 31 RT-qPCR primer synthesis information

gene number	Primers	Sequence (5'to3')	TM
11	*Col14a1*-F	CTGGACAGCCTGGATATTG	63
	Col14a1-R	GCTTCTTGCTCATCTTGGA	
12	*Clca4*-F	TCACAAGTTCCACCTCTTC	60
	Clca4-R	GGTCATAATCATCTCCTGGT	
13	*Fcer2*-F	CTGCTGACCTTGCTTCTC	63
	Fcer2-R	AAGTCCTTGGAAACCTGAG	
14	*Cacna1g*-F	ATCACCACCACTACCACTTG	60
	Cacna1g -R	ATTGGCATCCCTGTCCTG	
15	*Acad9*-F	CAGAACCCGACAGCAACT	63
	Acad9-R	GGACCTGGAGATTGGAACC	
16	*Fasn*-F	ACCTCAGTTATGGTGACCTC	60
	Fasn -R	GTAGGCAGTGGTGTAGACA	
17	*Awat2*-F	TGGAGGCTTCATCAATCG	63
	Awat2-R	ATGGTCGTTACAGGCTTAG	
18	*Aldh7a1*-F	CCGCTCCATACCAAACAAG	63
	Aldh7a1-R	TCCAGGACGATCCATAACC	
19	*Cyp1a1*-F	CAACCAAGAACTGCTTAG	63
	Cyp1a1-R	GCTGTAGAACCTCTCATT	
20	*Lnpep*-F	GCAGCGTGATTGGAGCATT	63
	Lnpep -R	ACAGCAACAGGAAACACTTGG	
21	*Wnk4*-F	ACGAAGGCTACTTGTCTCT	63
	Wnk4-R	GGAAGGAAGGAGGTCACT	
22	*Umod*-F	CTGTCCAGATGTTCCGATT	63
	Umod-R	GGTCTATGAAGCCTCCAC	
23	*Scnn1a*-F	CTCTATCAGTATGAGGAAGG	63
	Scnn1a-R	CCATCTCTTGGTTGTCAA	
24	*C1qc*-F	TGCCTGGTCTCTACTACT	63
	C1qc -R	CAAGTGGTCACAGAAGGT	
25	*Ptgs2*-F	TACCCTCCTACAGTCAAAG	60
	Ptgs2-R	CGTGGCATACATCATCAG	
26	*Adra1d*-F	ACAGTTCTACGCCAATCA	63
	Adra1d-R	AGCACCTCCTTCCTATGA	

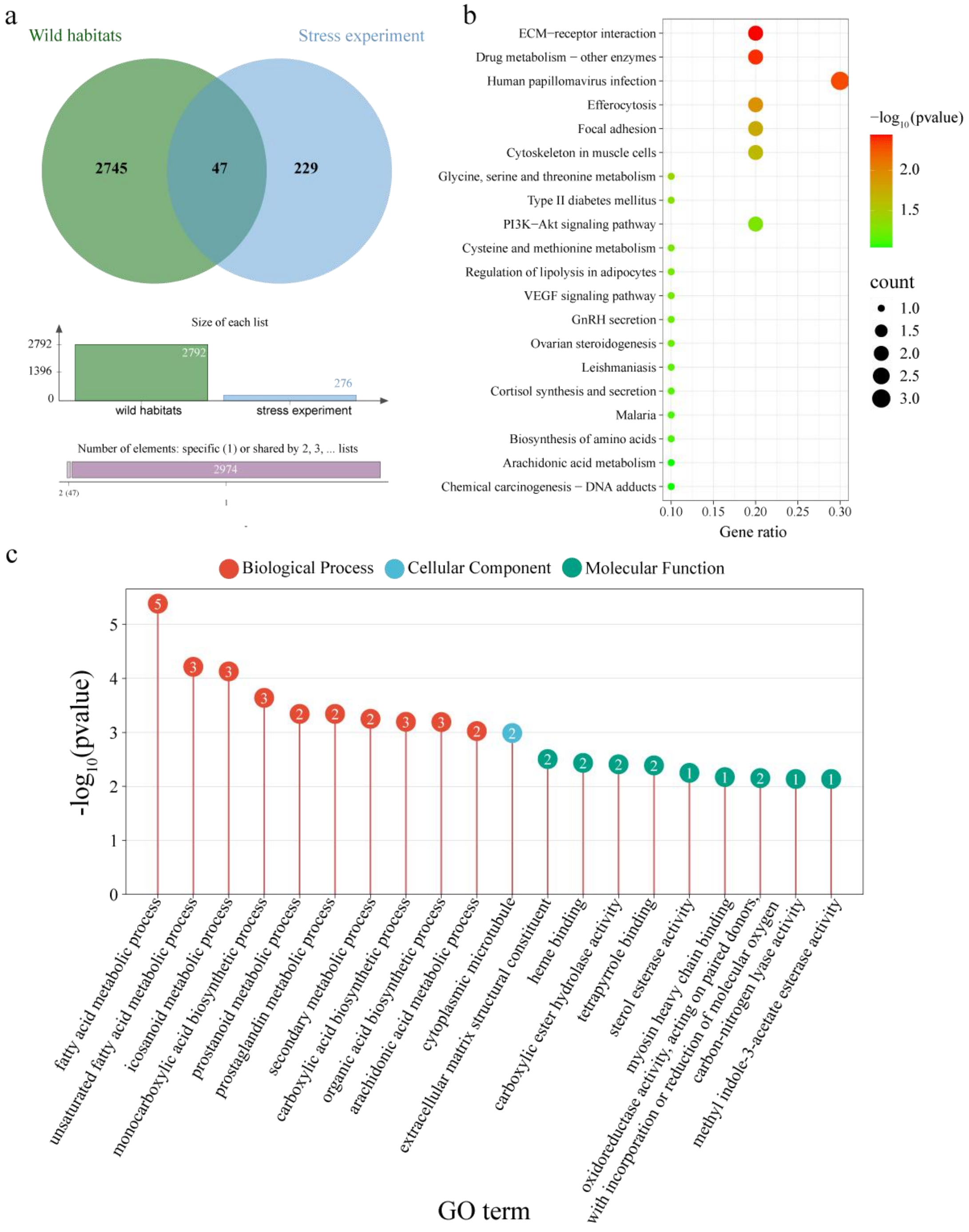

Supplementary Fig. 15 Venn diagram of differentially expressed genes in the wild environment and stress experiments, as well as the functional enrichment of intersecting genes. (a) Venn diagram of differentially expressed genes in the wild environment and stress experiments. (b) KEGG enrichment analysis of intersecting genes in the wild environment and stress experiments. (c) GO enrichment analysis of intersecting genes in the wild environment and stress experiments.

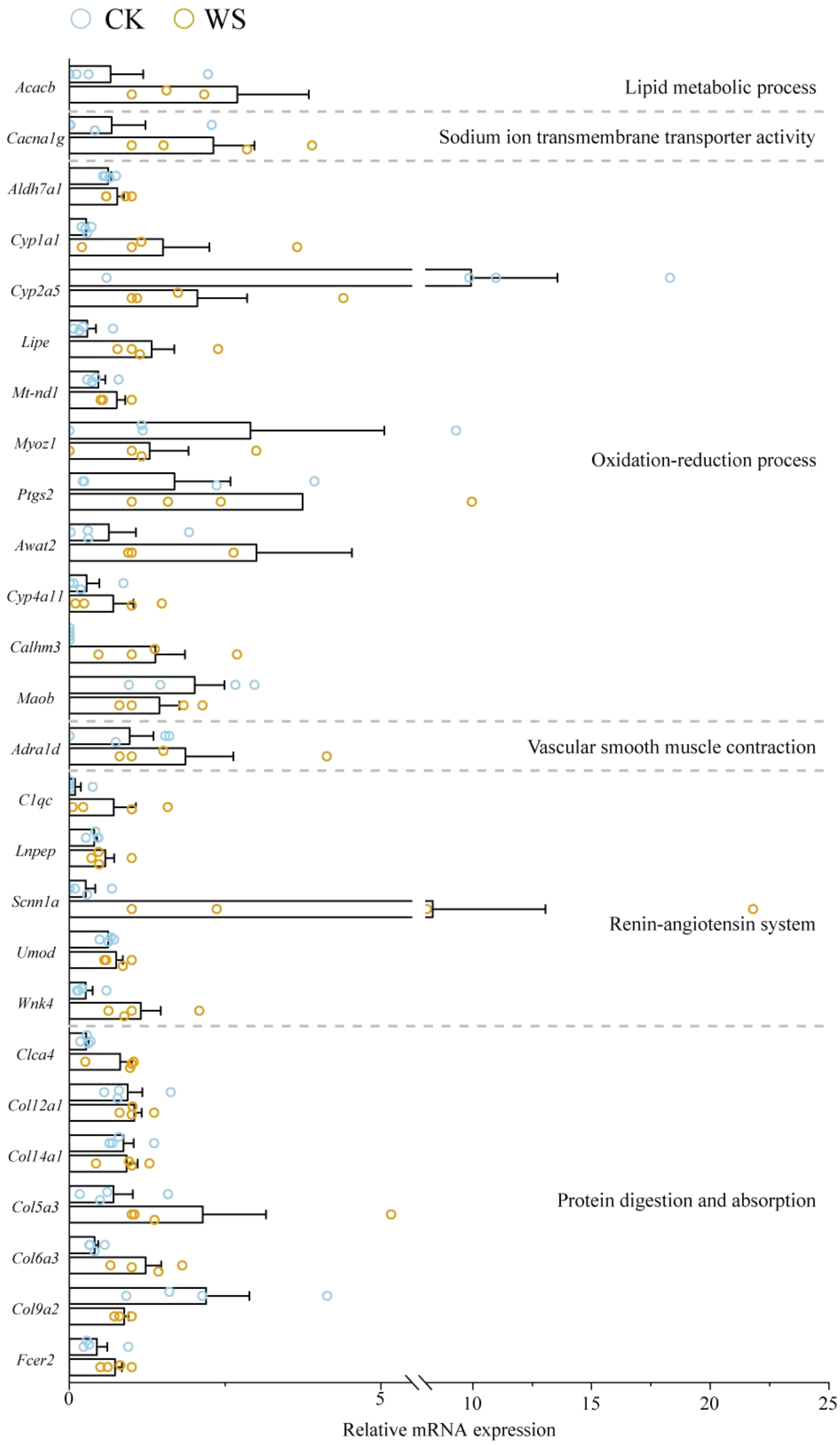

Supplementary Fig. 16 Relative mRNA expression of key differentially expressed genes (DEGs) in Siberian jerboa (CK vs. WS) detected through RT-qPCR

Note: CK, control group; WS, water deprivation stress.
